# Copper-Catalyzed Homocoupling of Boronic Acids: A Focus on B-to-Cu and Cu-to-Cu Transmetalations

**DOI:** 10.3390/molecules27217517

**Published:** 2022-11-03

**Authors:** Aude Salamé, Jordan Rio, Ilaria Ciofini, Lionel Perrin, Laurence Grimaud, Pierre-Adrien Payard

**Affiliations:** 1 Laboratoire des Biomolécules (LBM), Département de Chimie, Ecole Normale Supérieure, PSL University, Sorbonne Université, CNRS, 75005 Paris, France; 2 Université de Lyon, Université Claude Bernard Lyon I, CNRS, INSA, CPE, UMR 5246, ICBMS, 1 rue Victor Grignard, 69622 Villeurbanne, France; 3 École Nationale Supérieure de Chimie de Paris, Centre National de la Recherche Scientifique, Institute of Chemistry for Life and HealthSciences, PSL Research University, 75006 Paris, France

**Keywords:** transmetalation, Cu-catalysis, cross-coupling, homocoupling, mechanism, DFT, cyclic voltammetry, boronic acid

## Abstract

Controlling and understanding the Cu-catalyzed homocoupling reaction is crucial to prompt the development of efficient Cu-catalyzed cross-coupling reactions. The presence of a coordinating base (hydroxide and methoxide) enables the B-to-Cu(II) transmetalation from aryl boronic acid to Cu^II^Cl_2_ in methanol, through the formation of mixed Cu-(μ-OH)-B intermediates. A second B-to-Cu transmetalation to form bis-aryl Cu(II) complexes is disfavored. Instead, organocopper(II) dimers undergo a coupled transmetalation-electron transfer (TET) allowing the formation of bis-organocopper(III) complexes readily promoting reductive elimination. Based on this mechanism some guidelines are suggested to control the undesired formation of homocoupling product in Cu-catalyzed cross-coupling reactions.

## 1. Introduction

Metal-catalyzed coupling reactions are a staple of organic synthesis as they allow the controlled extension of the carbon backbone of molecules. One of the first historically described coupling reactions was reported by Ullman at the beginning of the last century (1901) to couple two identical aryl halides (homocoupling) in the presence of a stoichiometric amount of Cu(0) [1,2,3,4]. While Pd-catalyzed cross-coupling reactions are among the most used nowadays, Cu catalysis proved to be a nice complement [5] and is particularly interesting to catalyze C-C and C-heteroatom bond formations [5,6,7,8,9,10,11,12,13,14,15,16,17,18,19,20].

**Scheme 1:**
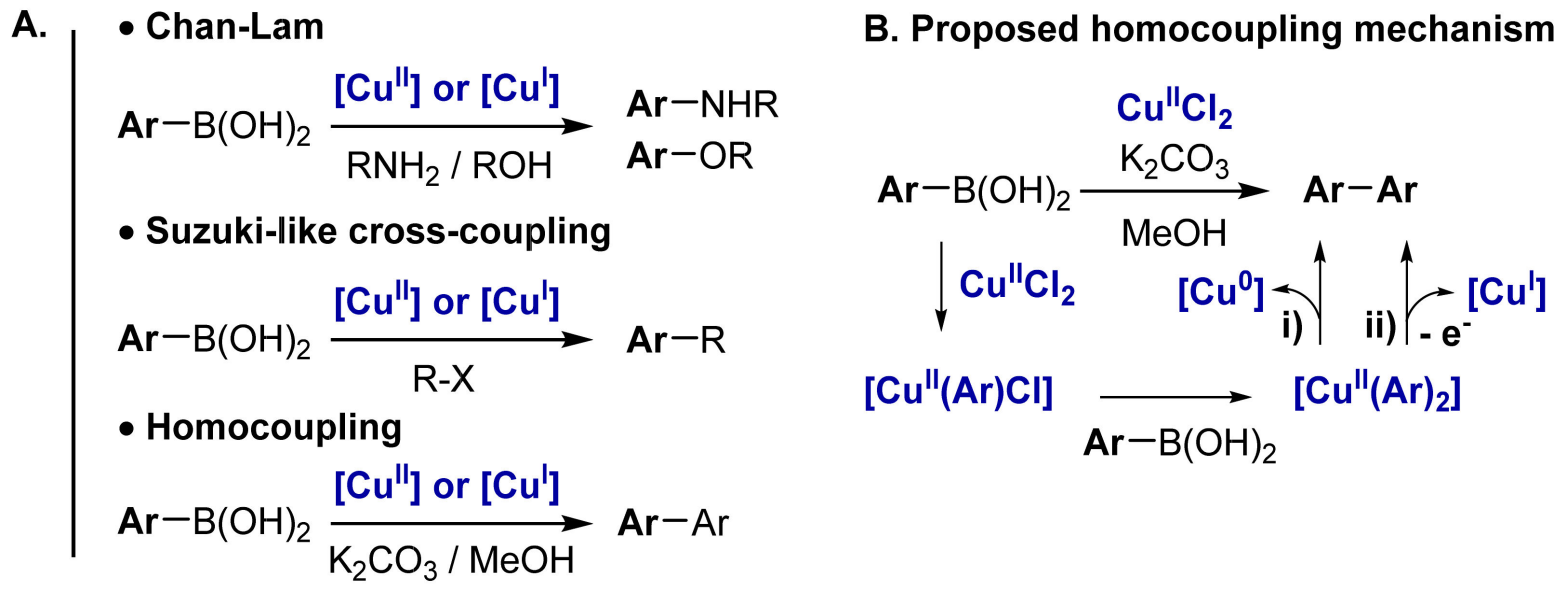
(**A**) Cu-catalyzed homo- and cross-coupling of aryl-boronic acids. (**B**) Currently proposed mechanisms of the Cu-catalyzed homocoupling of arylboronic acid (ArB(OH)_2_) in the presence of a base and current state-of-art catalytic cycle involving two successive B-to-Cu transmetalations and subsequent reductive elimination [6,7,8,9,21]. See Scheme 3 for the updated mechanism including the conclusion of the present work.

In the last decade, several protocols have been disclosed describing that mild conditions and low catalyst-loadings can perform the homocoupling of boronic acids using Cu salts (Scheme 1A) [6,7,8,9]. Cu-catalyzed homocoupling of boronic acids takes place at room temperature in the presence of a weak base such as K_2_CO_3_ (Scheme 1A) using Cu^II^Cl_2_ or Cu^I^Cl as catalyst precursors [7].

Controlling and understanding the Cu-catalyzed homocoupling reaction is of high interest (i) to enable efficient formation of symmetrical biphenyl compounds under mild conditions but also (ii) to disfavor this process in the case of Cu-catalyzed cross-coupling reactions. Deciphering the mechanism of homocoupling reactions is thus crucial to prompt the development of efficient Cu-catalyzed Cham-Lan [22,23,24,25,26] or Cu-catalyzed Suzuki-liked cross-couplings (Scheme 1A) [10,11]. By analogy with the established mechanism for Pd-catalyzed oxidative coupling of boronic acids [27,28], it was proposed that the Cu-catalyzed homocoupling of boronic acid takes place through two successive transmetalations on Cu(II) followed by (i) direct reductive elimination to yield Cu(0) or (ii) one-electron oxidation into Cu(III) and subsequent reductive elimination to yield Cu(I) (Scheme 1B) [6,7,8,9,21]. However, there is no evidence to support these mechanisms. Moreover, the role played by the base was not fully clarified. Indeed, starting from Cu(I), no base is required for the reaction to proceed [15], but the addition of a base is essential starting from Cu(II) [7]. Herein, we report evidence for an alternative homocoupling reaction pathway involving successive B-to-Cu and Cu-to-Cu transmetalation steps.

The mechanism of the Cu^II^Cl_2_-catalyzed homocoupling reaction of arylboronic acids in methanol under inert atmosphere was investigated experimentally using a combination of NMR, cyclic voltammetry, conductimetry pH-metry, and kinetics monitoring. The experimental insights were complemented by the exploration of reaction pathways computed at the DFT level. In particular, the effect of the added base and the involvement of higher-order Cu intermediates are clarified.

## 2. Results and Discussion

### 2.1. Cu-Catalyzed Homocoupling Reaction: Effect of Cu on Selectivity

**Scheme 2:**
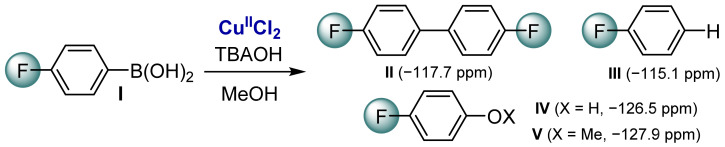
Stoichiometric reaction of [Cu^II^Cl_2_] with (*p*-F-C_6_H_4_)B(OH)_2_ (**I**) in methanol in the presence of tetrabutylammonium hydroxide (TBAOH). The reaction yields to the formation of the homocoupling product **II**, the proto-demetallation product **III**, and the oxidation products **IV** and **V**.

The stoichiometric reaction of Cu^II^Cl_2_ (10 mM) with 2 equiv of *para*-fluorophenylboronic acid (*p*-F-C_6_H_4_)B(OH)_2_ (**I**) was monitored using ^19^F NMR in MeOH (Scheme 2). The NMR tube was prepared under inert atmosphere to avoid the oxidative regeneration of Cu(II) from reduced Cu complexes and, thereof, the subsequent oxidative coupling (Figure S1). In MeOH, in the absence of an added base, (*p*-F-C_6_H_4_)B(OH)_2_ (−112.2 ppm) is stable and no decomposition reaction was observed after 5 h at r.t. (Figure S2).

Tetrabutylammonium hydroxide (TBAOH) was used as a model base for this reaction since it is soluble in methanol and allows us to separate the effect of the base from the ones related to a coordinating counter-cation. In the presence of 20 equiv of (*p*-F-C_6_H_4_)B(OH)_2_ and 12 equiv of TBAOH, the signal of the boronic acid reagent (**I**) decreased rapidly and several new signals appeared at −117.7, −115.1, −126.5, and −127.9 ppm. They correspond to the homocoupling product **II**, the product of the boronic acid protodemetalation **III**, the phenol **IV**, and the ether **V** oxidation products of the boronic acid, respectively (Scheme 2 and Figure S3). All signals were attributed by addition of a small amount of each pure product in the NMR tube.

Since the boron derivative is stable in the absence of Cu^II^Cl_2_, even in the presence of a base (Figure S4), protodemetalation is expected to be mediated by Cu. Consequently, the formation of a protodemetalation product is indicative of B-to-Cu transmetalation followed by protonation, while the formation of homocoupling product comes from transmetalation and subsequent reductive elimination.

The ratio of protodemetalation vs. homocoupling products was shown to be highly dependent on the Cu^II^Cl_2_ and added base concentrations. At high Cu^II^Cl_2_ concentration (20 mM), no protodemetalation product was detected, while at low concentration about 20% of the protodemetalation product was observed (Figure S5). This indicates that the kinetics of the homocoupling reaction follows a higher reaction order with respect to Cu(II) than the kinetics of the protodemetalation reaction. This suggests the possible involvement of multinuclear Cu(II) complexes. The concentration of TBAOH was shown to impact the final product distribution: a high base concentration (0.8 equiv with respect to PhB(OH)_2_) favors the proto-demetallation (Figure S6).

### 2.2. Cyclic Voltammetry Monitoring of the Homocoupling Reaction

**Figure 1:**
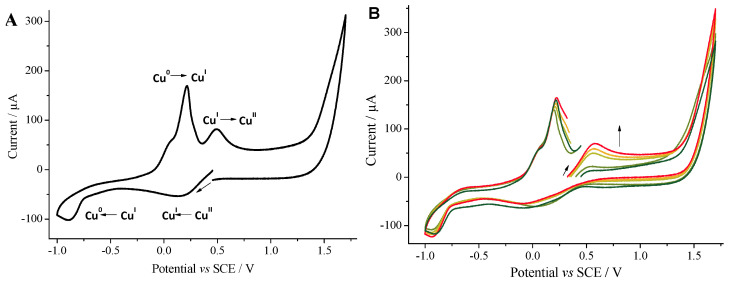
(**A**). Cyclic voltammogram toward reduction potentials of a solution of Cu^II^Cl_2_ (2 mM) in MeOH (see Figure S9 for the voltammogram toward oxidation). (**B**). Cyclic voltammogram toward oxidation potentials monitoring the reduction in Cu(II) to Cu(I) by PhB(OH)_2_ in the presence of TBAOH Cu^II^Cl_2_ (2 mM, dark green). Addition of PhB(OH)_2_ (10 mM, light green). Addition of TBAOH (5 mM, apple green) at t = 0. After 20 min (light orange). After 30 min (orange). After 45 min (red). Working electrode: glassy carbon (Ø = 3 mm); scan rate: 0.5 V s^−1^; supporting electrolyte: *n*Bu_4_NBF_4_ (0.3 M); recorded at ambient temperature starting at the open circuit potential (OCP).

Electrochemical analysis methods were used to monitor the structure of ligandless Cu species under reaction conditions. Cyclic voltammetry is a powerful tool to access the oxidation state of a metal in solution [29] and, consequently, to scrutinize the oxidation state of a catalyst during oxidative or reductive chemical steps [27]. Cu^II^Cl_2_ (2 mM) is characterized in methanol (Figure 1A) by two reduction peaks at 0.25 and −0.75 V vs. the standard calomel electrode (SCE). They respectively correspond to the reduction to Cu(I) and subsequent deposition of Cu(0) at the electrode. The deposited Cu(0) is re-oxidized to Cu(I) at 0 and 0.2 V vs. SCE and Cu(I) to Cu(II) at 0.5 V vs. SCE. The addition of phenylboronic acid does not modify the shape of the voltammogram (Figure S7).

Upon addition of TBAOH (6 equiv) in the presence of PhB(OH)_2_ (10 equiv), the reduction peak related to Cu(II) disappears progressively (Figure S8), while the oxidation peak Ox3, proportional to the concentration of Cu(I), is increasing (Figure 1B). This highlights the formation of Cu(I) and the consumption of Cu(II). In addition, since the reduction in Cu(I) can only originate from reductive elimination, the variation of the concentration of Cu(I) with time relates to the kinetics of the coupling reaction.

To gain more insights into the role played by Cu and the base in the coupling mechanism, we monitored the kinetics of Cu(I) formation using a rotating disk electrode (RDE) [30]. Using this set-up, the potential of the working electrode can be imposed at 0.7 V vs. SCE to enable Cu(I) oxidation. In this condition, the current intensity measured is proportionated to the concentration of Cu(I) in the solution. In the presence of an excess of phenylboronic acid and of base, all kinetic profiles could be fitted by a pseudo first-order law in Cu(I) (Figure S10).

**Figure 2:**
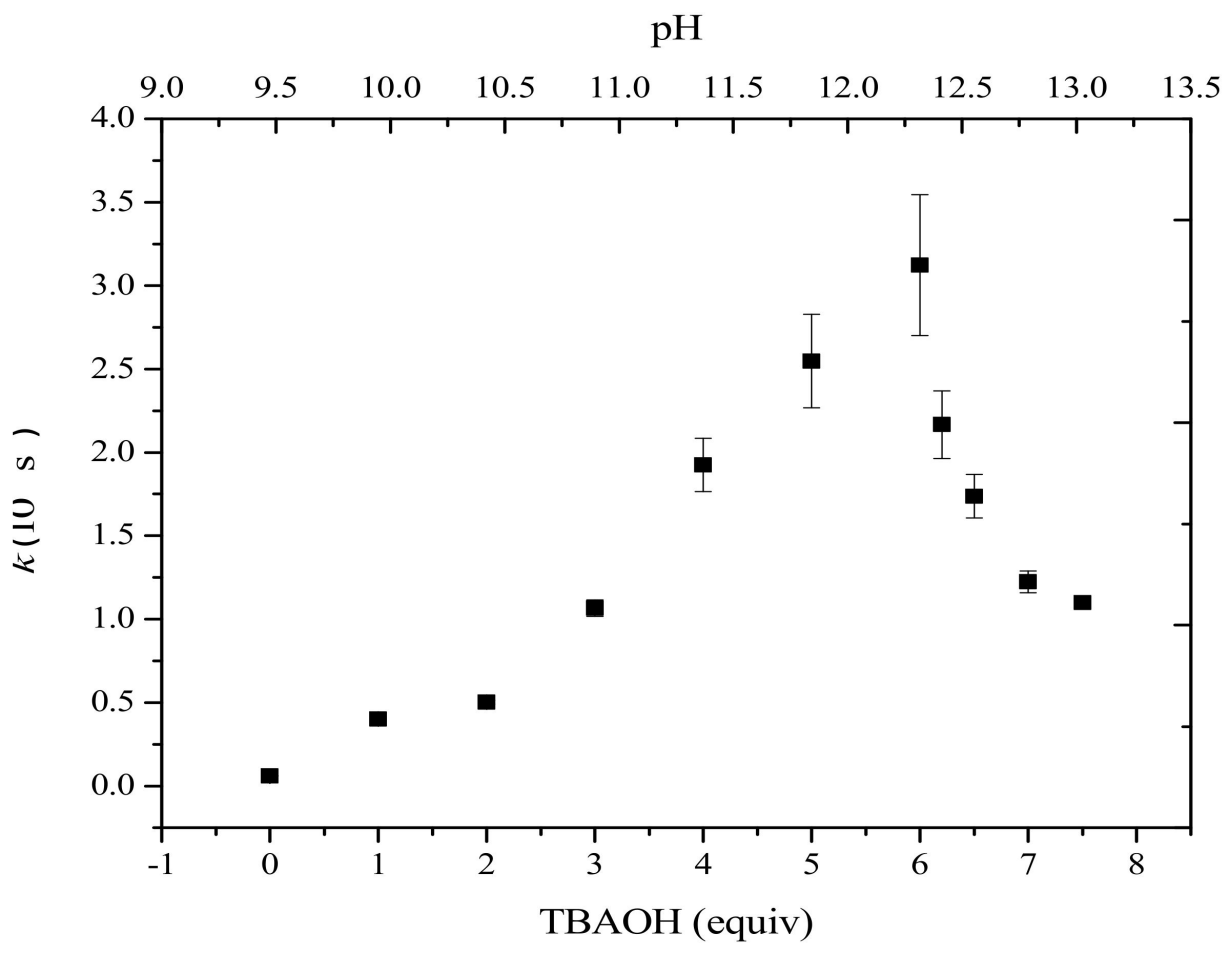
Variation of the pseudo first-order rate constant (*k*, s^−1^) of the formation of Cu(I) as estimated by fitting the measured electric current intensity at the polarized rotating electrode (+0.7 V/SCE) as a function of the initial concentration of TBAOH. For each point of the bell-shaped distribution, four experiments were repeated and the standard deviation was estimated (error bars).

The variation of the apparent rate constant for the formation of Cu(I) with increasing pH is depicted in Figure 2. At low base concentration, the rate of the coupling reaction increased linearly with the concentration of added base, while at higher concentration (6 to 8 equiv), the rate of the reaction decreased sharply. By analogy with the proposed transmetalation mechanism, this bell-shape behavior could be indicative of the formation of a μ-OH-bridged Cu-(μ-OH)-B pre-transmetalation intermediate [31]. In the absence of base, the reaction is apparently limited because of the absence of OH groups, while at high concentration the predominance of Cu hydroxide and boronate species likely hamper the formation of mixed complexes between Cu and B.

### 2.3. Electronic Effects on the Kinetics of the Reaction: Hammett Plot

**Figure 3:**
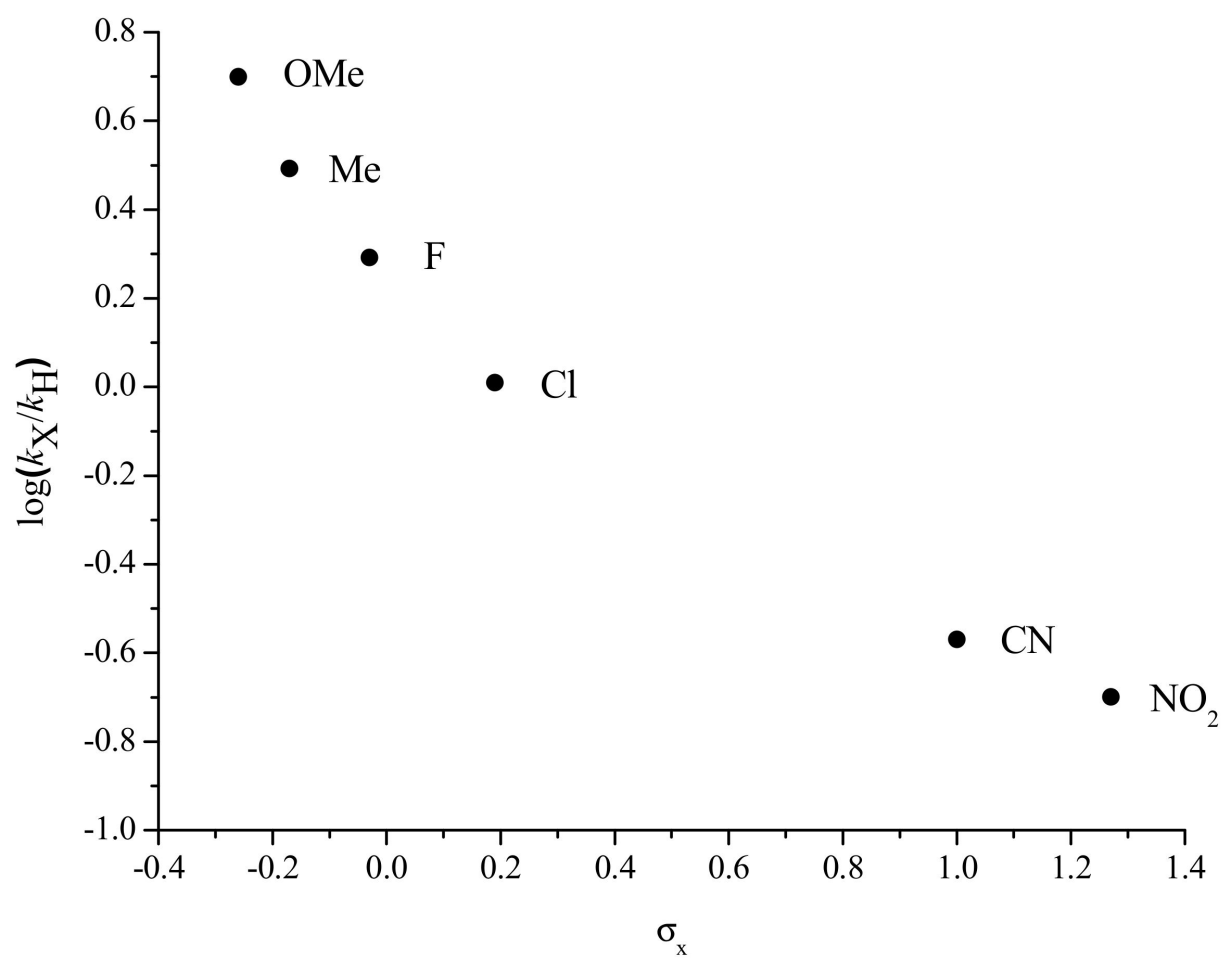
Hammett plot for the reduction in Cu(I) (1 mM) by *para*-substituted phenylboronic derivatives (*p*-X-C_6_H_4_)B(OH)_2_ (10 mM) (X = MeO, Me, F, Cl, CN and NO_2_) in the presence of TBAOH (8 mM) (see Figure S11 for the fit of the associated kinetics); *k*_H_ pseudo first-order rate constant for X = H, *k*_X_ pseudo first-order rate constant for a substituent X.

To get more information related to the electronic effects over the rate-determining state (RDS) of the overall coupling process, the kinetics of the Cu(I) formation was monitored for a series of *para*-substituted phenylboronic acid derivatives. The variations of the pseudo first-order rate constant were obtained at pH = 11 and 12. In both cases, electron-rich boronic acid derivatives react faster than electron-poor ones (Figure 3 and Figure S11). The negative slope of the Hammett plot for the reaction is thus indicative of a decrease in the partial charge on the aromatic ring during the RDS. This is consistent with transmetalation being the rate-determining step. The nonlinearity of the Hammett plot drawn can be indicative of a shift in pre-equilibrium or a change of the RDS step [32].

### 2.4. Structure of Cu(II) in Methanol

To interpret the effect of the base concentration on the kinetics of the coupling reaction, we attempted to gather more information regarding the structure of Cu^II^Cl_2_ in MeOH solution as a function of the pH. The conductivity of the Cu^II^Cl_2_ electrolyte in MeOH was studied as a function of the square root of the concentration (Figure S12). The nonlinear plot obtained is typical of a weak electrolyte resulting from a limited dissociation of Cu^II^Cl_2_ into CuCl^+^ and Cl^−^ in MeOH, in contrast to the behavior reported in water [33]. This agrees with the much lower permittivity of MeOH compared to water [34].

As PhB(OH)_2_ is a weak acid-in-methanol solution accordingly to Equation (1) (pKa_(MeOH)_ (PhB(OH)_2_/PhB(OH)_3_^−^) = 11.2, see Figure S13), the pH of the methanol solution can be controlled by adding sub-stoichiometric amounts of TBAOH to PhB(OH)_2_. Thereof, the resulting mixture of boronate PhB(OH)_3_^−^ and boronic acid acts as a pH buffer.

PhB(OR)_2 (MeOH)_ + ROH _(MeOH)_ = PhB(OR)_3_^−^_(MeOH)_+ H^+^_(MeOH)_; R = H, Me (1)

**Figure 4:**
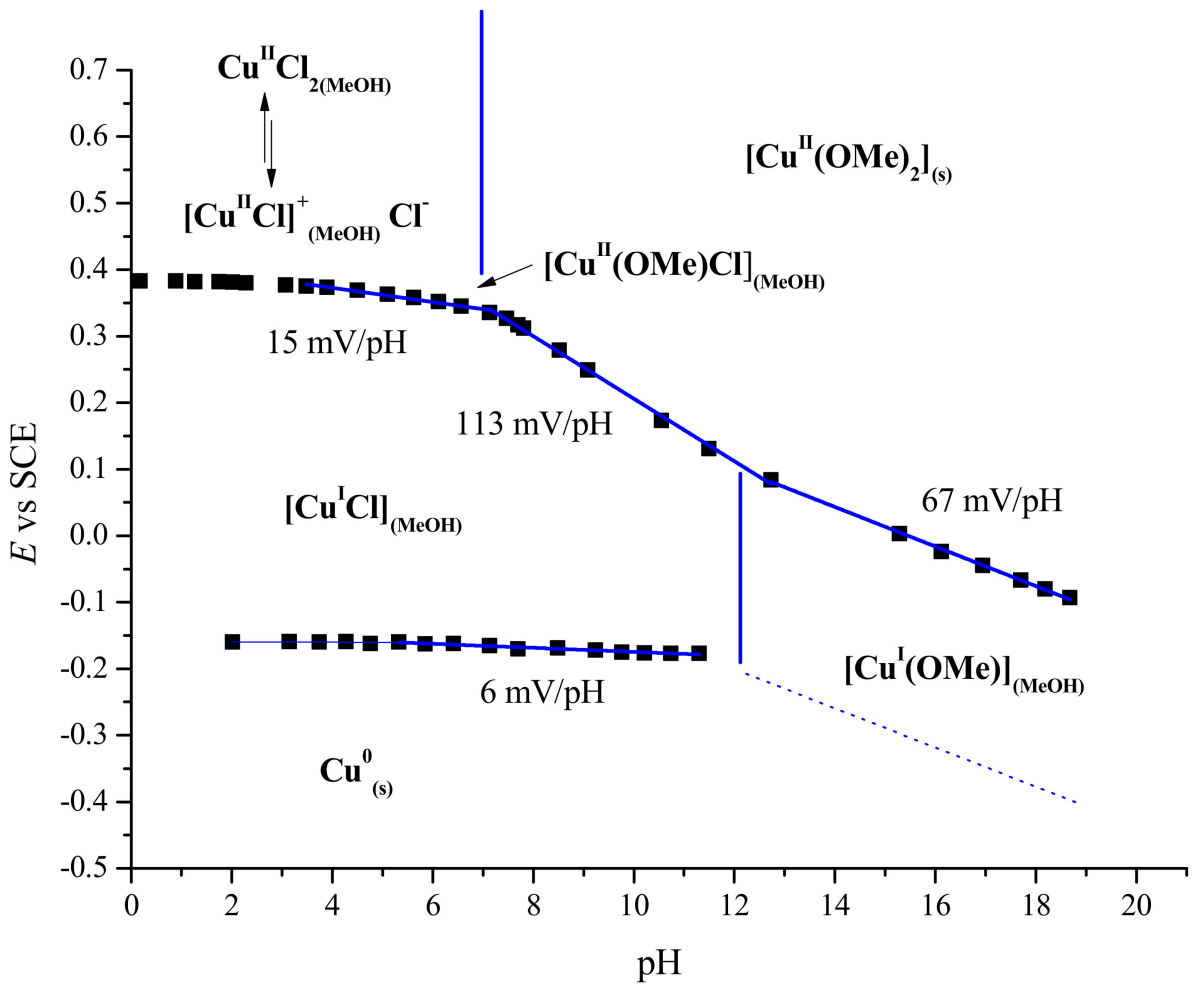
Pourbaix diagram of Cu(I) and Cu(II) in MeOH (Cu(I) and Cu(II) concentration 2 mM).

The [PhB(OH)_3_^−^]/[PhB(OH)_2_] concentration ratios and the associated pH range that enable the coupling reaction to proceed (Figure 2 and Figure S13) were estimated between pH 10.5 and 12.5. Under these conditions, the number of anionic ligands on Cu was determined using the Pourbaix diagram of Cu^II^Cl_2_ in MeOH. This diagram was constructed by measuring the open-circuit potential (OCP) of (i) an equimolar solution of Cu^I^Cl and Cu^II^Cl_2_ (2 mM/2 mM, top plot) at a Pt working electrode and (ii) a Cu(0) working electrode immersed in a 2 mM solution of Cu(I), as a function of the pH of the solution, which was varied by the addition of a small quantity of a 1 M solution of TBAOH in MeOH (Figure 4).

For pH ranging from 4 to 8, the potentials of the Cu(II)/Cu(I) and Cu(I)/Cu(0) couple show very little variation; this indicates that counter anions are not coordinated to either Cu(I) or Cu(II). Above pH 8, the Cu(II)/Cu(I) curve shows a nonlinear transition that is indicative of the partial formation of either [Cu^II^(OR)Cl] and/or [Cu^II^(OR)]^+^ complexes (pKa_1_ (CuCl_2_/Cu(OH)Cl) = 9). This transition is also indicated by the formation of green complex. Above pH 10, the slope of the later increases to a value of 113 mV/pH; this corresponds to the coordination of anionic ligands to Cu(II) to form the [Cu^II^(OR)_2_] (R = H, Me) alkoxide complexes that partially precipitate (pKa_2_ (CuCl_2_/Cu(OR)_2_ = 16.3). Of note, such a precipitate was never observed under the reaction conditions, even at a higher base concentration. For pH above 11, [Cu^I^(OH)] becomes the predominant species for Cu(I).

### 2.5. Computational Mechanistic Investigation: B-to-Cu(II) Transmetalation

Based on the results described in Section 2.4, Cu(II) is likely to exist under the form of a [Cu^II^(OR)_2_] complex, with OR^−^ = HO^−^, MeO^−^, PhB(OH)_3_^−^ depending on the reaction conditions, for pH ranging between 9.5 and 12.5. However, at low pH, the formation of [Cu^II^Cl(OR)] or [Cu^II^(OR)]^+^ cannot be excluded. Thereof, the ability of all these species to promote B-to-Cu(II) transmetalation was evaluated by means of a computational mechanistic investigation performed at the DFT level using the B3LYP hybrid functional [35,36,37,38]. Hereafter, energies refer to Gibbs energies (∆*G*, kcal mol^−1^) estimated at 298 K and 1 atm upon full geometry optimization. Solvation by methanol has been modeled by means of a micro-solvation approach [39] that combines an explicit description of the first solvation sphere and an implicit representation of the bulk solvation effects by a polarization continuum model [40]. The fully detailed computational approach is available in Section 4, Materials and Methods. In the following sections, DFT-optimized structures are tagged by Arabic numerals. If the complex is cationic, a + is added (e.g., **5^+^**). For neutral complexes, the counter anion is specified by a superscript (e.g., **4^OH^**). The presence and the number of explicit solvent molecules are indicated by a superscript n = (e.g., **4^+,n=2^**), ranging from 0 to 2. Finally, when spin intercrossing occurs, i.e., in Section 2.6, the spin state (singlet *s*, doublet *d*, triplet *t*) of the complex is specified in superscript brackets (e.g., **9^MeO(t),n=0^**). Transition states are indicated using the prefix **TS-**.

**Figure 5:**
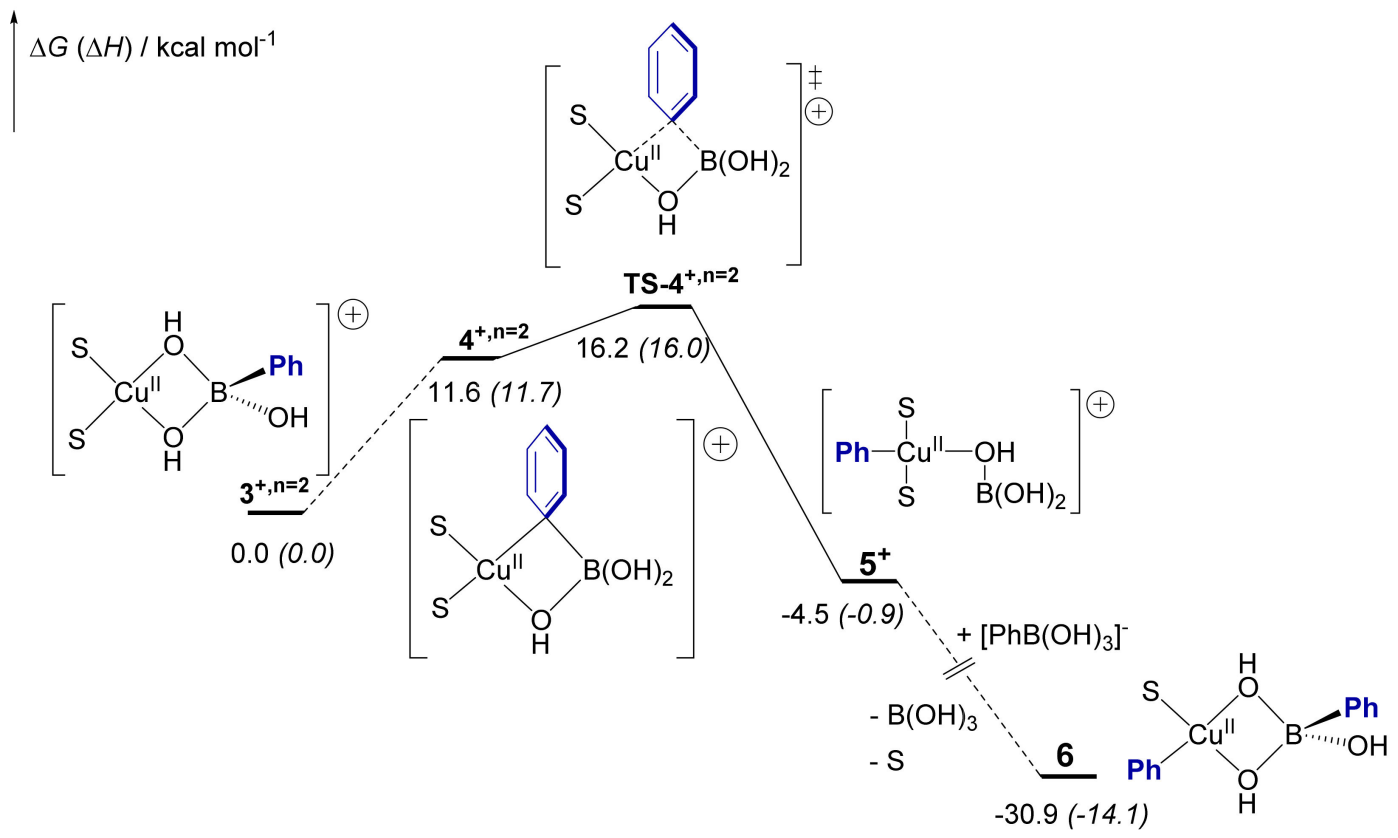
Reaction profile of the Cu-to-B transmetalation in the heterobimetallic cationic intermediate [(S)_2_Cu(μ-OH)_2_B(Ph)(OH)]^+^ (**3^+,n = 2^**) (S = MeOH) computed at the DFT level. ∆*G* (∆*H*) are given in kcal mol^−1^. See Figures S14–S16 for alternative pathways.

The transmetalation mechanism starting from variously solvated heterobimetallic adducts [Cu^II^(PhB(OH)_3_)(MeOH)_n_]^+^ (**3^+,n^**) between phenylboronate (**1**) and [Cu^II^MeOH_2_]^2+^ (**2**) was investigated. Figure 5 depicts the most relevant Gibbs energy profile in the case of the di-solvated [Cu^II^(PhB(OH)_3_)(MeOH)_2_]^+^ (**3^+,n = 2^**) heterobimetallic adduct. The influence of solvation on the stabilities of **3^+,n^** (n = 0, 1 or 2) and on the B-to-Cu transmetalation energy barriers is discussed in the SI (Figure S15) [39].

**Figure 6:**
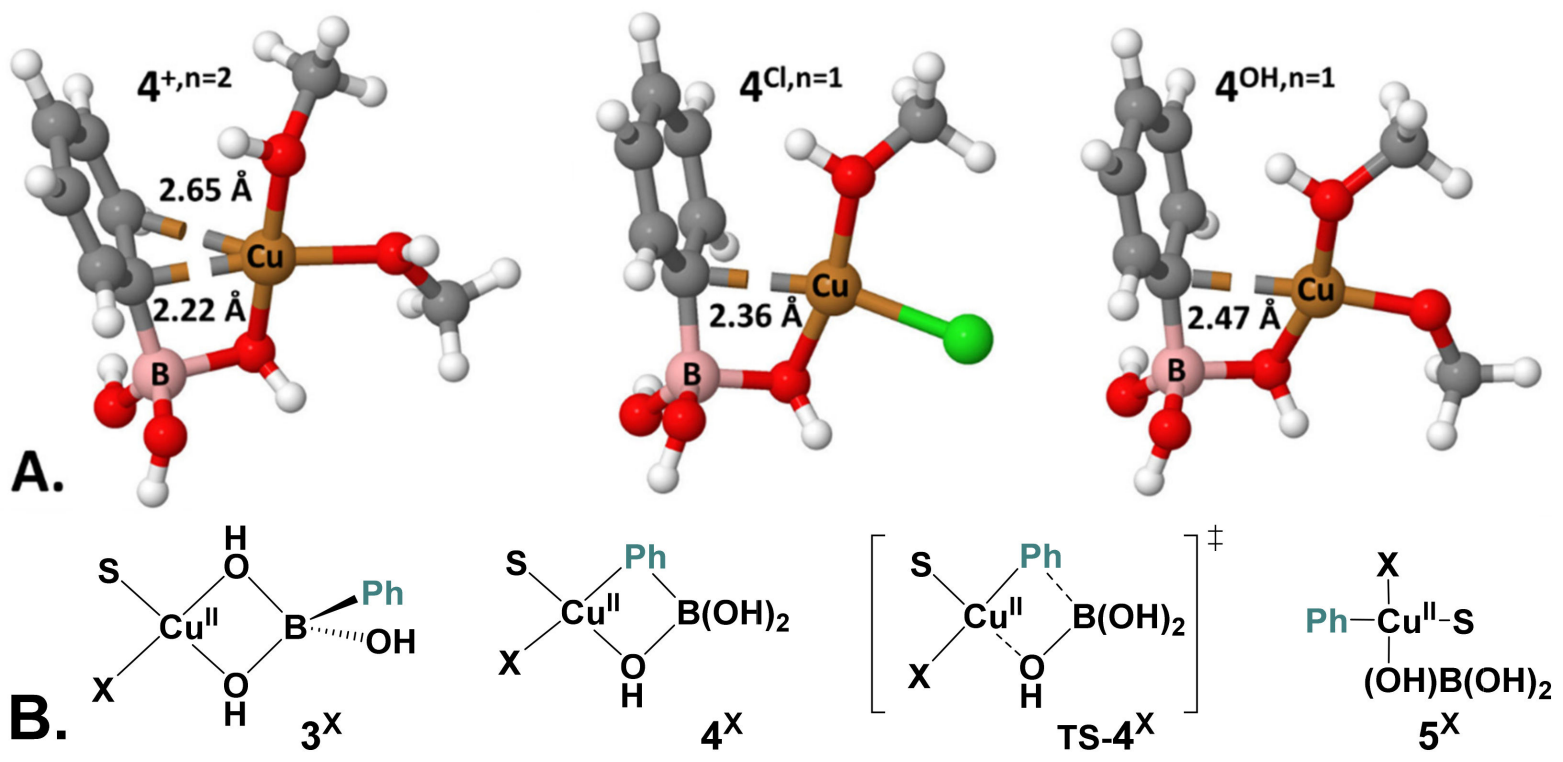
(**A**). 3D structures of optimized intermediates **4^X,n^** (X = +, Cl or MeO, n = 1 or 2). H in white, C in grey, O in red, B in pink, Cu in orange, and Cl in green. (**B**). Structures of intermediates and transition states involved in the B-to-Cu transmetalation (see Table 1 for the associated energies). See Figures S15 and S16 for alternative solvation states.

Complex **3^+,n = 2^** features two bridging μ-OH groups between Cu(II) and B atoms and the four-membered ring atoms are nearly co-planar (Φ_Cu-O-B-O_ = 1.5°, Figure 6A). The formation of such a mixed Cu-(μ-OH)-B dimer as a key pre-transmetalation species mirrors previous reports on Pd [41,42,43] or Ni complexes [44]. Starting from complex **3^+,n=2^**, one of the bridging hydroxy groups is exchanged with the phenyl group via a torsion along the non-exchanging B-µ-O bond. This isomerism is endergonic by 11.6 kcal mol^−1^ and gives the pre-transmetalation intermediate **4^+,n=2^**. In **4^+,n = 2^**, the interaction between Cu and the Ph moiety has a pronounced σ character, as indicated by the open B-C*^ipso^*-C*^para^* angle of 171° and the short Cu-C*^ipso^* distance (2.22 Å) (Figure 6A). This pre-activation allows the easy transfer of the Ph group via the early transition state **TS-4^+,n = 2^**. Relative to **3^+,n = 2^**, transmetalation via **TS-4^+,n = 2^** requires us to overcome an overall Gibbs energy barrier of 16.2 kcal mol^−1^ to yield the transmetalated complex *trans*-[Cu^II^(Ph)(MeOH)_2_(μ-OH)B(OH)_2_]^+^ (**5^+^**). This is in good agreement with previous calculations related to boronate transmetalation reported on a parent Cu system [45]. From **3^+,n = 2^** to **TS-4^+,n=2^**, the main contribution to the energy barrier is the formation of pre-complex **4^+,n = 2^**. From cationic complex **5^+^**, coordination of a second phenylboronate molecule [PhB(OH)_3_]^−^ (**1**) along with the release of boronic acid leads to intermediate **6**. The large exergonicity of the reaction and the overall Gibbs energy barrier of ca. 16 kcal mol^−1^ are in line with experimental data.

Alternative pathways of transmetalation have been explored starting from neutral monosolvated Cu(II) complexes [Cu^II^(MeOH)(X)(μ-OH)_2_B(Ph)(OH)], X = Cl (**3^Cl,n = 1^**), MeO (**3^MeO,n = 1^**). The Gibbs energies of the computed profiles are gathered in Table 1. The presence of a chloride bounded to Cu merely affects the overall transmetalation barrier (17.2 kcal mol^−1^) compared to the cationic system (16.2 kcal mol^−1^). By comparison, methoxide is slowing down transmetalation as indicated by an overall energy barrier of 23.7 kcal mol^−1^. This could be one possible explanation for the observed pH dependence of the homocoupling kinetics.

The lower ability to transmetalate to hydroxo- or methoxy-copper complexes is also reflected in the structure of the pre-transmetalation intermediate **4^X,n = 1^**. Both **4^OH,n = 1^** and **4^MeO,n = 1^** show very little σ-character of the Cu-C bond with bond length of 2.4 Å and flat B-C^ipso^-C par angle of 175°. This is related to a lower donation from Cu to Ph in agreement with the higher partial charge at the Cu in **TS-4^MeO,n = 1^** (1.326 |e^−^|) compared to **TS-4^Cl,n = 1^** (1.256 |e^−^|) (Table S1). Finally, the formation of transmetalation products **5^X^** is either endergonic (X = Cl) or isergonic (X = OH and MeO).

Based on these considerations, we have assessed the influence of solvent coordination on the energy barrier of the transmetalation step (Figures S15 and S16). Mono-solvated **3^MeO,n = 1^** is merely more stable than the solvent-free **3^MeO,n=0^** (∆G = −0.7 kcal mol^−1^); both are likely in equilibria in MeOH solution. The formation of the pre-complex **4^MeO,n = 0^** is facilitated compared to the mono-solvated case (+9.5 kcal mol^−1^) and the energy of the subsequent transmetalation transition state **TS-4^MeO,n = 0^** is 4.7 kcal mol^−1^ lower in energy than the one computed for the mono-solvated transition state **TS-4^MeO,n = 1^** (∆G^‡^ = 18.0 vs. 22.7 kcal mol^−1^, Figure S15). These specific solvent effects on transmetalation make the use of an explicit solvent model mandatory for the description of such systems. Indeed, the dynamics of solvation plays an important role in slowing down or facilitating the transmetalation, as suggested for other related systems [46,47,48].

The mechanism presented above accounts for the experimental data regarding the transmetalation but does not rationalize the formation of homocoupling products. One possible mechanism to explain the homocoupling involves a second B-to-Cu transmetalation of the phenyl group followed by a reductive elimination (Scheme 1). The energy profile of this reaction pathway were computed starting from [Cu^II^(MeOH)(PhB(OH)_3_)Ph] (**6**) (see Figure S17). Activation energies related to a second transmetalation have been computed to at least 25.1 kcal mol^−1^. Based on the experimental conditions and the kinetic measurements, this reaction sequence cannot thereof account for the formation of homocoupling products, though it is commonly suggested.

### 2.6. Computational Mechanistic Investigation: Cu(II)-Cu(II) Transmetalation and Reductive Elimination

**Figure 7:**
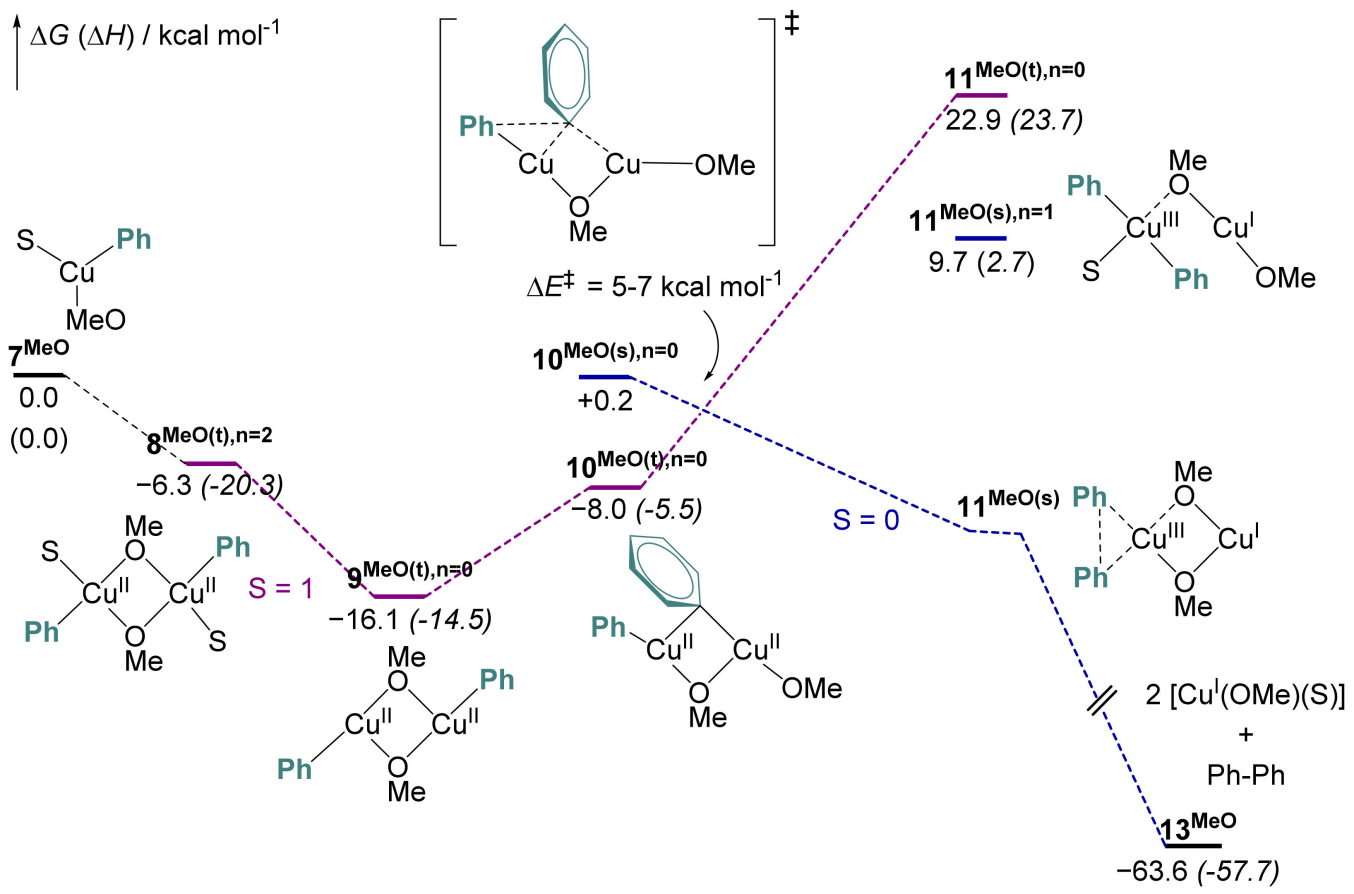
Gibbs energy profile of the Cu-to-Cu second transmetalation and subsequent reductive elimination computed at the DFT level (see the computational details). **∆*G*** (∆*H*) are given in kcal mol^−1^.

To rationalize the formation of homocoupling products, we have considered alternative reaction pathways in which the second transmetalation takes place between two copper centers. Such a mechanism would support the higher kinetic order with respect to Cu experimentally inferred for the homocoupling reaction. In the following section, we will only discuss the reaction sequence starting from the monomeric PhCu(S)OMe (**7^MeO^**) that results from the first transmetalation (Figure 7).

The second transmetalation starts with the dimerization of **7^MeO(d)^** in its doublet state (S = 1/2) to form the high-spin di-solvated Cu^II^-Cu^II^ dimer **8^MeO(t),n = 2^** (S = 1), whose formation is computed exergonic by 6.3 kcal mol^−1^. The release of two solvent molecules from **8^MeO(t),n = 2^** leads to an even more stable dimer triplet complex **8^MeO(t),n = 0^** (∆*G* = −16.1 kcal mol^−1^ relative to **7^MeO(d)^**). Torsion along the nonreactive Cu^II^-(μ-OH) bond enables the exchange between one bridging μ-MeO group and the Ph ring to yield **9^MeO(t),n = 0^** in its triplet state. This step is endergonic by 8.1 kcal mol^−1^. More details on the speciation of these intermediates can be found in Tables S2 and S3. For this set of dimers (**8** to **9^MeO(t),n = 0^**), the closed-shell singlet state corresponding to the mixed-valence dimer Cu(III)-Cu(I) is always computed higher in energy.

The triplet Cu(II)-Cu(II) complex **9^MeO(t),n = 0^** can then undergo Cu-Cu transmetalation to yield the mixed-valence (Cu(III)-Cu(I)) singlet complex **10^MeO(s),n = 0^**. This latter is not a true minimum on the potential energy surface and spontaneously undergoes reductive elimination to yield Cu(I)-Cu(I) complex **11^MeO(s),n = 0^** and biphenyl (Figure S18). Since this transformation requires a spin transition, the associated singlet and triplet energy surface were scanned to estimate the activation energy of this process. The electronic energy of activation for the transmetalation and coupled electron transfer (TET) was estimated to 5 and 7 kcal mol^−1^. In the case of the monosolvated **10^MeO,n = 1^**, the barrier for the TET process is nearly doubled and the product of transmetalation complex **11^MeO(s),n = 1^** is an energy minimum (Figure S18). Note that direct reductive elimination at monomeric species Cu(II) is kinetically prohibited (Figure S17). This mechanistic picture for reductive elimination is in agreement with results recently reported by Casares on the role of electron transfer between organocopper complexes in transmetalation reactions [49].

## 3. Conclusions

**Scheme 3:**
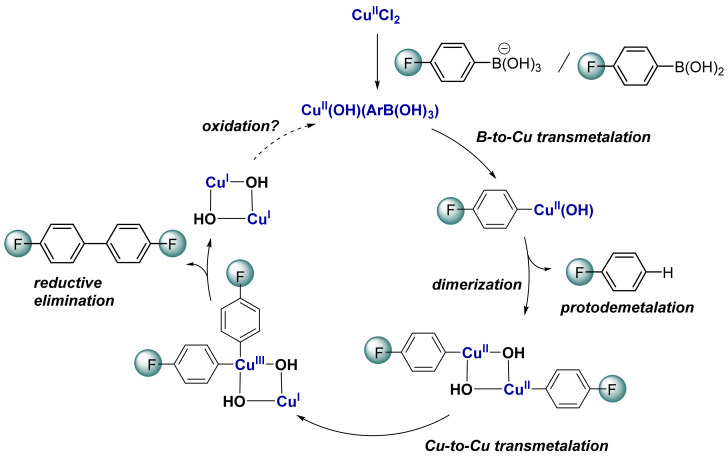
Updated mechanism proposed for Cu(II)-catalyzed homocoupling of arylboronic acids. See Figure S19 for the most favored pathway for the complete homocoupling process.

The presence of a coordinating base (hydroxide and methoxide) enables the first B-to-Cu(II) transmetalation from aryl boronic acid to Cu^II^Cl_2_ in methanol, while an excess of base is detrimental (Scheme 3). This suggests the formation of heterobimetallic Cu-(μ-OH)-B intermediates as previously suggested [45,50] and as confirmed by our DFT calculations. In contrast with previous suggestions from the literature, a second B-to-Cu transmetalation to form bis-aryl Cu(II) complexes is kinetically prevented [6,7,9]. Alternatively, the formation of organocopper(II) dimers is favored and the latter can undergo a coupled transmetalation-electron transfer (TET) allowing the formation of a mixed-valence organocopper(III)/copper(I) complex able to perform reductive elimination.

The possibility to catalyze homocoupling reactions using Cu(I) in the absence of a base suggests that transmetalation proceeds either via Cu(I) oxidation by O_2_ and the associated generation of OH^−^ [51,52]:

2 Cu^+^ + 1/2 O_2_ + H_2_O = 2 Cu^2+^ + 2 OH^−^

or through a mechanism involving a Cu(I) oxo complex as proposed in the case of Pd(0) or via another oxidation intermediate [27,28]. This point is currently under study within our group.

Based on mechanistic insights herein reported, we can tentatively propose some guidelines to control the undesired formation of homocoupling product in the context of Cu-catalyzed cross-coupling reactions. The pH should be controlled and optimized for each catalytic system to favor transmetalation and prevent the side reactions. The use of bidentate ligands is expected to disfavor the formation of dimeric Cu(II) species responsible for homocoupling. Alternatively, using a supported Cu catalyst or working at low catalyst loadings may also be helpful. Finally, the control of the oxygen concentration in the media is crucial to limit the formation of Cu(II) species in Cu-catalyzed non-oxidative cross-coupling reactions.

## 4. Materials and Methods

Structure optimizations, energy estimation, and NBO 6 analyses [53] were performed with the gaussian 09 software [54]. Molecules have been optimized using an ultrafine grid as implemented in gaussian. The Becke hybrid functional, B3LYP functional was used for all calculations [35,36,37,38]. The 6–31+G augmented double-ζ Pople’s basis sets were used for B, C, and H atoms, and complemented with an extra polarization function for O atom. Quasi relativistic Stuttgart–Dresden 10MWB electron core pseudopotentials and their associated basis sets were used for Cu and Cl atoms [55]. Since the reaction occurs in a highly polar solvent, particular attention was paid to model the solvation. The most favorable solvation states of key intermediates have been verified (see the supporting information), and the bulk solvation effect has been represented by a continuum model for all calculations [56]. Unless specified, optimized transition states were checked by analytical frequency calculations and their connectivity were verified by IRC following.

## Figures and Tables

**Table 1:** Transmetalation paths starting from neutral heterobimetallic Cu(II)/B complexes [Cu^II^(MeOH)_n_(X)(μ-OH)_2_B(Ph)(OH)] (**3^X,n^**), X = Cl, MeO, n = 0 or 1. ∆*G* (∆*H*) in kcal mol^−1^.

X, n	3^X^	4^X^	TS-4^X^	5^X^
Cl, n = 1	0.0 (0.0)	9.9 (11.4)	17.2 (18.5)	0.0 (3.7)
MeO, n = 1	0.0 (0.0)	11.9 (11.8)	23.7 (22.7)	6.5 (8.5)
MeO, n = 0	0.0 (0.0)	9.5 (19.1)	18.0 (16.9)	0.6 (−3.8)

## Data Availability

The data that support the findings of this study are available in the Figures or Supplementary Materials of this article.

## Supplementary Materials

The following supporting information can be downloaded at: https://www.mdpi.com/article/10.3390/molecules27217517/s1, **Figure S1** ^19^F{^1^H} kinetic monitoring of the reaction of *p*-FPhB(OH)_2_ (0.2 M) and Cu^II^Cl_2_ (20 mM, 20 mol%) in MeOH in the presence of K_2_CO_3_ (0.2 M, 1 equiv). A solution of *n*Bu_4_BF_4_ 0.1 M in d^6^-DMSO contained in a sealed capillary was used as an internal standard (signal at −148.5 ppm). As indicated on the zoomed insert the formation of the homocoupling product proceed very fast when the base is added and a plateau is obtained around 0.01 mol L^−1^ corresponding to a stoichiometry of two Cu(II) *per* mol of homocoupling product formed. **Figure S2** ^19^F{^1^H} NMR spectrum of a solution of *p*-FPhB(OH)_2_ (20 mM, 2 equiv) and Cu^II^Cl_2_ (10 mM) in MeOH in the absence of added base **A.** at t = 0 h, **B.** after 5h. A solution of *n*Bu_4_BF_4_ 0.1 M in d^6^-DMSO contained in a sealed capillary was used as an internal standard (signal at −148.5 ppm). **Figure S3** ^19^F{^1^H} NMR spectrum of a solution of *p*-FPhB(OH)_2_ (20 mM, 20 equiv), TBAOH (12 mM, 12 equiv) and CuCl_2_ (1 mM, 1 equiv) in MeOH. A solution of fluorobenzene in d^6^-DMSO contained in a sealed capillary was used as an internal standard (signal at −113.1 ppm). fluorobenzene in MeOH (−115.2 ppm), homocoupling product (−117.9 ppm), *p*-F-phenol (−126.5 ppm) and *p*-F-anisole (−128.1 ppm). **Figure S4** ^19^F{^1^H} NMR spectrum of a solution of *p*-FPhB(OH)_2_ (20 mM) and TBAOH (1 equiv) in the absence of Cu^II^Cl_2_ in MeOH at t = 0 and t = 24 h. The signal of [*p*-FPhB(OH)_3_]^−^ is detected at −121.6 ppm.**Figure S5** ^19^F{^1^H} NMR spectrum of a solution of *p*-FPhB(OH)_2_ (20 mM, 1 equiv), TBAOH (12 mM, 0.5 equiv) and CuCl_2_ (20 mM, 1 equiv) in MeOH. A solution of fluorobenzene in d^6^-DMSO contained in a sealed capillary was used as an internal standard (signal at −113.1 ppm).**Figure S6** ^19^F{^1^H} NMR spectrum of a solution of *p*-FPhB(OH)_2_ (20 mM, 10 equiv) and Cu^II^Cl_2_ (2 mM) in MeOH in the presence of **a)** 4 equiv of TBAOH, **b)** 8 equiv of TBAOH. A solution of fluorobenzene in d^6^-DMSO contained in a sealed capillary was used as an internal standard (signal at −113.1 ppm). **Figure S7** Cyclic voltammogram toward reduction (left) and oxidation (right) potentials of a solution of CuCl_2_ (2 mM) in MeOH (red line) and in the presence of PhB(OH)_2_ (10 mM) (green line). Working electrode: glassy carbon (Ø = 3 mm); scan rate: 0.5 V s^−1^; supporting electrolyte: nBu_4_BF_4_ (0.3 M); recorded at ambient temperature starting at the Open Circuit Potential (OCP). **Figure S8** Cyclic voltammetry toward reduction potentials monitoring of the reduction of Cu(II) (CuCl_2_, 2 mM) to Cu(I) by PhB(OH)_2_ in the presence of TBAOH (Dark green). After addition of PhB(OH)_2_ (10 mM, Light green). (Apple green) Addition of TBAOH (5 mM) at t = 0. (Light orange) After 20 min. (Orange) After 30 min. (Red) After 45 min. Working electrode: glassy carbon (Ø = 3 mm); scan rate: 0.5 V s^−1^; supporting electrolyte: nBu_4_BF_4_ (0.3 M); recorded at ambient temperature starting at the Open Circuit Potential (OCP). **Figure S9** Cyclic voltammogram toward oxidation of a solution of CuCl_2_ (2 mM) in MeOH. Working electrode: glassy carbon (Ø = 3 mm); scan rate: 0.5 V s^−1^; supporting electrolyte: nBu_4_BF_4_ (0.3 M); recorded at ambient temperature starting at the Open Circuit Potential (OCP). **Figure S10** Kinetic monitoring of the reduction of CuCl_2_ (1 mM) by PhB(OH)_2_ (10 mM, 10 equiv) in the presence of TBAOH (6 mM, 6 equiv). Working electrode: glassy carbon (Ø = 3 mm); rotation rate: 1000 min^−1^, imposed potential +0.7 V/SCE; supporting electrolyte: nBu_4_BF_4_ (0.3 M); thermostat 20 °C, recorded at ambient temperature. **Figure S11** Kinetic monitoring of the reduction of CuCl_2_ (1 mM) by *p*-X-PhB(OH)_2_ (10 mM, 10 equiv, X = MeO, CH_3_, F, Cl, CN et NO_2_) in the presence of TBAOH (4 mM, left / 8 equiv right). Working electrode: glassy carbon (Ø = 3 mm); rotation rate: 1000 min^−1^, imposed potential +0.7 V/SCE; supporting electrolyte: nBu_4_BF_4_ (0.3 M); thermostat 20 °C, recorded at ambient temperature. **Figure S12 A.** Evolution of the conductivity (σ, mS m^−1^) versus the concentration of added Cu^II^Cl_2_ (mol m^3^). **B.** Evolution of the ionic molar conductivity of the solution λ (mS m^2^ mol^−1^) versus the square root of the concentration of added Cu^II^Cl_2_ (mol^1/2^ m^−3/2^)_._ **Figure S13** Evolution of the pH during the titration of a solution of phenylboronic acid (10 mmol L^−1^) by TBAOH (1.0 mol L^−1^ in MeOH). The pKa of the couple PhB(OH)_2_/PhB(OH)_3_^−^ can be read 0.5 equiv, pKa = 11.2. **Figure S14** Reaction profiles of the Cu-to-B transmetalation starting for (i) the heterobimetallic cationic complex [(S)_2_Cu(μ-OH)_2_B(Ph)(OH)]^+^ (**3^+^**) (ii) the neutral heterobimetallic intermediate complexes [S(X)Cu(μ-OH)_2_B(Ph)(OH)] (**3^X^**) for X = Cl, OH and MeO, computed at the DFT level. Free energies and enthalpies ∆*G* (∆*H*) are given in kcal mol^−1^ **Figure S15** Gibbs free energy calculated at the DFT level for the first boron-to-copper transmetalation involving cationic (red) and neutral (X = MeO, green) intermediates for different solvation states *(i.e. n = 0 to 2 MeOH coordinated to Cu atom)*. Free energies and enthalpies ∆*G* (∆*H*) are given in kcal mol^−1^. **Figure S16** Gibbs free energy calculated at the DFT level for the first boron-to-copper transmetalation involving cationic neutral (X = Cl, n = 0 to 2) intermediates for different solvation states. Free energies and enthalpies ∆*G* (∆*H*) are given in kcal mol^−1^. **Figure S17** Reaction profiles of the second Cu-to-B transmetalation and following reductive elimination on monomeric organocopper starting for organocopper heterobimetallic complex [(S)(Ph)Cu(μ-OH)_2_B(Ph)(OH)] (**6**) computed at the DFT level. Free energies and enthalpies ∆*G* (∆*H*) are given in kcal mol^−1^. **Figure S18** Scans of the triplet and singlet potential energy surfaces over the (µ-(Ph)C_sp2_)-Cu^2^ distances for Cu^II^-Cu^II^ transmetalation *A:* without any MeOH coordinated, *B:* with a “trapping” MeOH and *C:* over the Cu^1^-(µ-(Ph)C_sp2_)-Cu^2^ angle starting from complexes **11^Cl(t)^**. **Figure S19** Complete mechanism for homocoupling process computed at the DFT level. Free energies and enthalpies ∆*G* (∆*H*) are given in kcal mol^−1^. **Table S1**. Comparison of B-to-Cu transition states. Dihedral angles are in degree, distances are in Å, partial charge in atomic unit (1 a.u. = |e^−^|). The numbers indicated between parenthesis correspond to the bond length variation between **4^X,n^** and **TS-4^X,n^** (X = MeOH, Cl or MeO). **Table S2**. Formation free energy of Cu^II^-Cu^II^ dimers with R^1^, R^2^ = Cl, OH and MeO as bridging groups. Gibbs free energy (∆***G***, kcal mol^−1^) and enthalpies (*∆H,* kcal mol^−1^) are calculated using the monomers (R^1^CuPh) and (R^2^CuPh) as a reference. In some cases, one or more MeOH molecules are de-coordinates from Cu during optimization and these structures are quoted *n.d.* The formation of dimers is favored in basic media (^−^OH or ^−^OMe present). **Table S3**. Solvation of Cu^II^-Cu^II^ dimers with R^1^ = Ph as bridging groups. ∆***G*** *(∆H)* in kcal mol^−1^. In some cases, one or more MeOH molecules are de-coordinate from Cu during optimization and these structures are quoted *n.d.* Cartesians coordinates for all DFT-optimized structures are available in the Cartesians.xyz supplementary file.
