# Supplementary material for: Copper-Catalyzed Homocoupling of Boronic Acids: A Focus on B-to-Cu and Cu-to-Cu Transmetalations
Source: Molecules. 2022 Nov 3;27(21):7517. doi: 10.3390/molecules27217517 (PMC9653983; doi:10.3390/molecules27217517)
Supplement: Supplementary file 1 [file molecules-27-07517-s001.zip › Supporting_Information_VF.pdf]

Supporting Information

Copper-Catalyzed Homocoupling of Boronic Acids: A focus on Boron-to-Copper and Copper-to-Copper Transmetalation

Aude Salamé,[1,†] Jordan Rio [2,†] Ilaria Ciofini,[3,*] Lionel Perrin,[2,*]
Laurence Grimaud[1,*] and Pierre-Adrien Payard[1,2,*]

E-mail: ilaria.ciofini@chimieparistech.psl.eu; lionel.perrin@univ-lyon1.fr;
laurence.grimaud@ens.psl.eu; pierre-adrien.payard@univ-lyon1.fr

[†] These authors cotributed equally.

[1] Laboratoire des Biomolécules (LBM), Département de Chimie, Ecole Normale Supérieure, PSL University, Sorbonne Université, CNRS, 75005 Paris, France

[2] Université de Lyon, Université Claude Bernard Lyon I, CNRS, INSA, CPE, UMR 5246, ICBMS, F-69622 Villeurbanne cedex, France.

[3] École nationale supérieure de chimie de Paris, Centre national de la recherche scientifique, Institute of Chemistry for Life and HealthSciences, PSL Research University, Paris, France.

Table of contents

1. $^{19}F\{^1H\}$ monitoring of Cu(II)-catalyzed homocoupling reactions

2. Cyclic voltammograms and kinetics monitoring of the formation of Cu(I)

3. B-to-Cu(II) transmetalation

3.1. Pre-equilibria

3.2. NBO analyses of B-to-Cu transition states

4. Cu-to-Cu transmetalation

4.1 Speciation of Cu(II)-Cu(II) dimers.

4.2 Pathways for Cu-Cu transmetalation and reductive elimination.

1. $^{19}F\{^1H\}$ monitoring of the Cu(II)-catalyzed homocoupling reaction

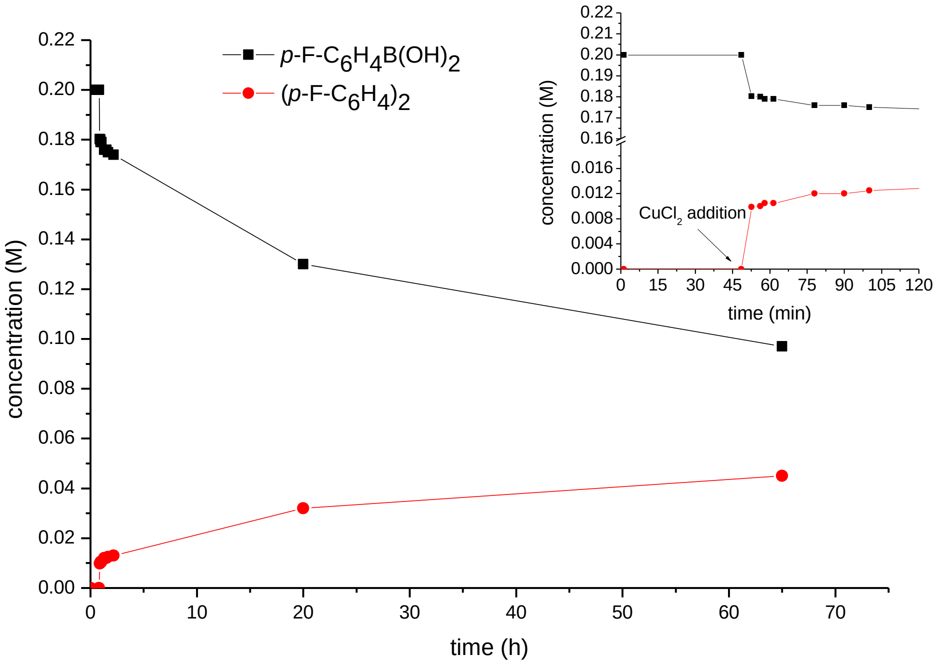

Figure S1. $^{19}F\{^1H\}$ kinetic monitoring of the reaction of *p*-FPhB(OH)$_2$ (0.2 M) and Cu^{II}Cl$_2$ (20 mM, 20 mol%) in MeOH in the presence of K_2CO_3 (0.2 M, 1 equiv). A solution of *n*Bu$_4$BF$_4$ 0.1 M in d^6-DMSO contained in a sealed capillary was used as an internal standard (signal at -148.5 ppm). As indicated on the zoomed insert the formation of the homocoupling product proceed very fast when the base is added and a plateau is obtained around 0.01 mol L^{-1} corresponding to a stoichiometry of two Cu(II) *per* mol of homocoupling product formed.

A.

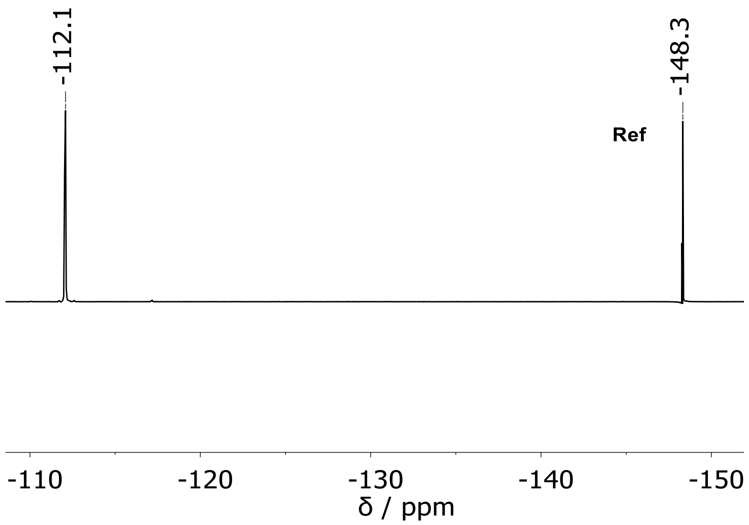

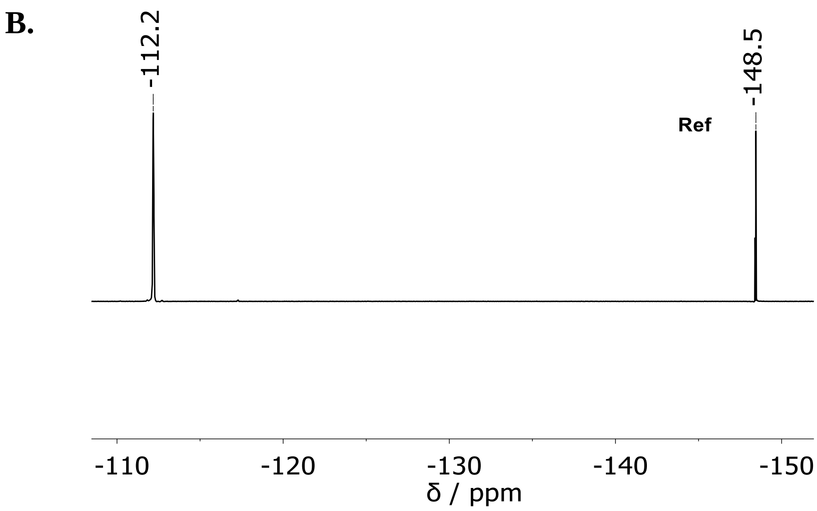

Figure S2. $^{19}F\{^1H\}$ NMR spectrum of a solution of *p*-$FPhB(OH)_2$ (20 mM, 2 equiv) and $Cu^{II}Cl_2$ (10 mM) in MeOH in the absence of added base **A.** at t = 0 h, **B.** after 5h. A solution of nBu_4BF_4 0.1 M in d^6-DMSO contained in a sealed capillary was used as an internal standard (signal at -148.5 ppm).

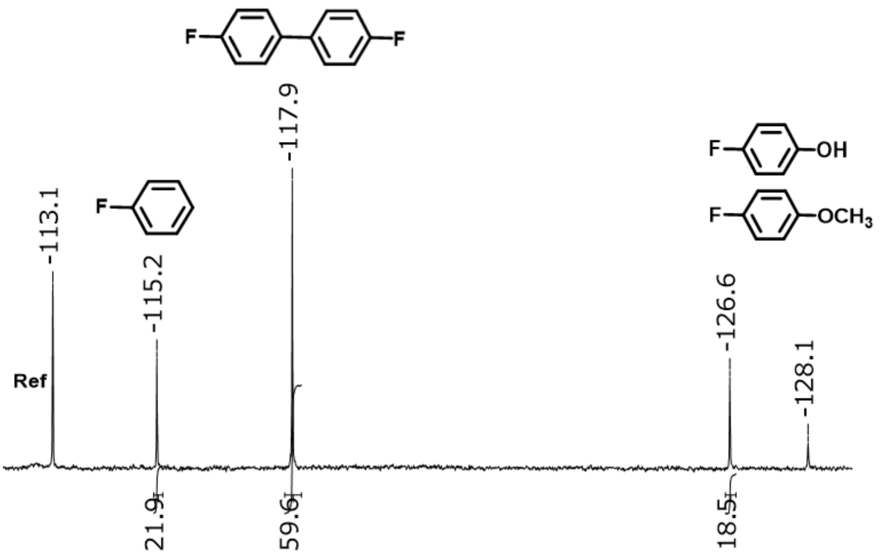

Figure S3. $^{19}F\{^1H\}$ NMR spectrum of a solution of *p*-$FPhB(OH)_2$ (20 mM, 20 equiv), TBAOH (12 mM, 12 equiv) and $CuCl_2$ (1 mM, 1 equiv) in MeOH. A solution of fluorobenzene in d^6-DMSO contained in a sealed capillary was used as an internal standard (signal at -113.1 ppm). fluorobenzene in MeOH (-115.2 ppm), homocoupling product (-117.9 ppm), *p*-F-phenol (-126.5 ppm) and *p*-F-anisole (-128.1 ppm).

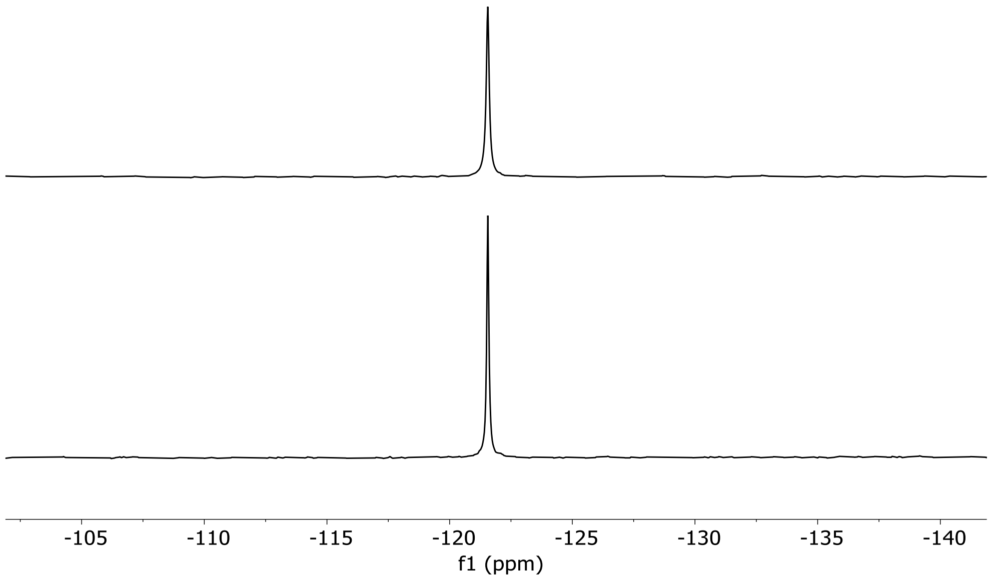

Figure S4. $^{19}F\{^{1}H\}$ NMR spectrum of a solution of *p*-FPhB(OH)$_2$ (20 mM) and TBAOH (1 equiv) in the absence of $Cu^{II}Cl_2$ in MeOH at t = 0 and t = 24 h. The signal of [*p*-FPhB(OH)$_3$]$^-$ is detected at -121.6 ppm.

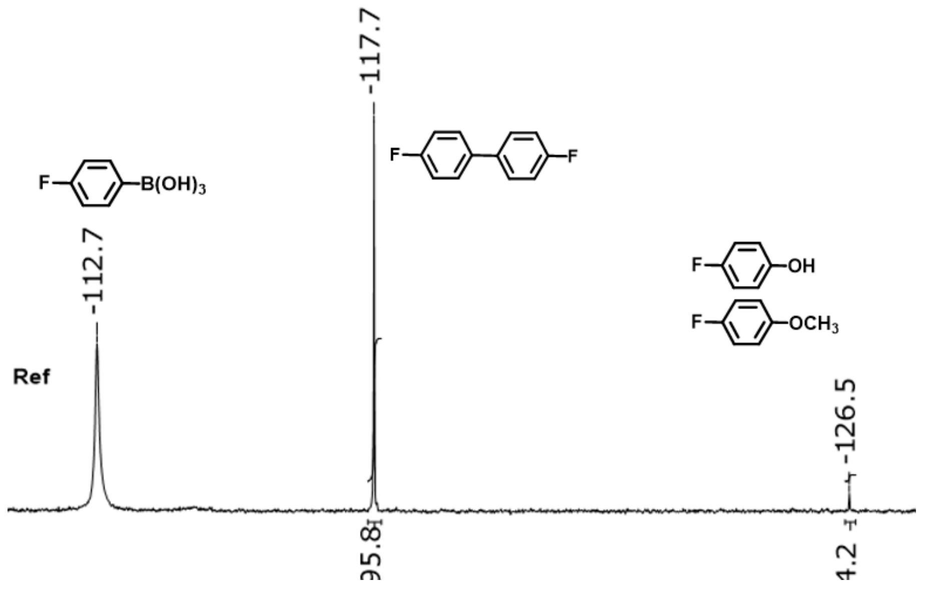

Figure S5. $^{19}F\{^{1}H\}$ NMR spectrum of a solution of *p*-FPhB(OH)$_2$ (20 mM, 1 equiv), TBAOH (12 mM, 0.5 equiv) and $CuCl_2$ (20 mM, 1 equiv) in MeOH. A solution of fluorobenzene in d^6-DMSO contained in a sealed capillary was used as an internal standard (signal at -113.1 ppm).

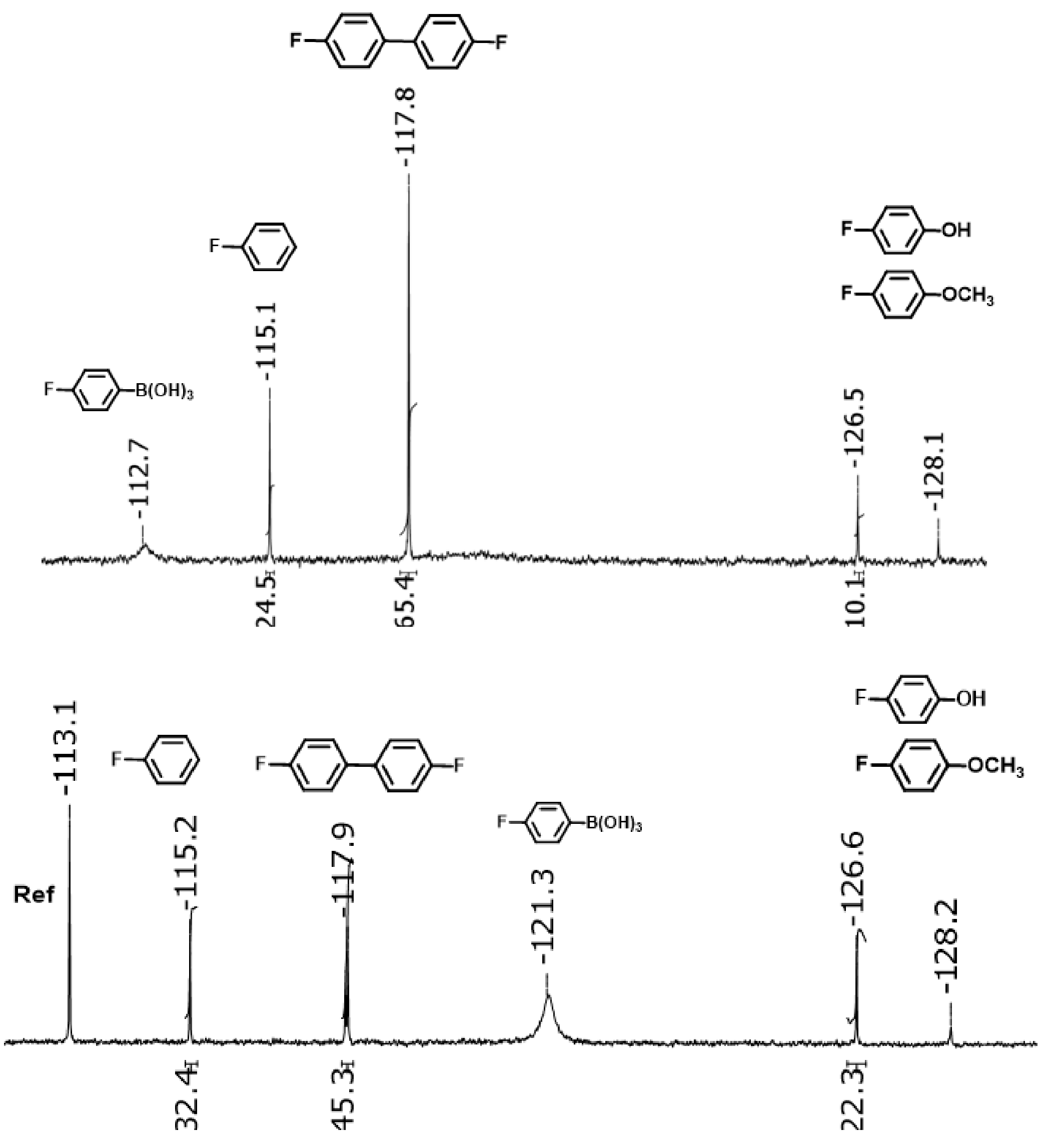

Figure S6. $^{19}F\{^{1}H\}$ NMR spectrum of a solution of *p*-FPhB(OH)$_2$ (20 mM, 10 equiv) and $Cu^{II}Cl_2$ (2 mM) in MeOH in the presence of **a)** 4 equiv of TBAOH, **b)** 8 equiv of TBAOH. A solution of fluorobenzene in d^6-DMSO contained in a sealed capillary was used as an internal standard (signal at -113.1 ppm).

2. Cyclic Voltammograms and Kinetic Monitoring of the Formation of Cu(I)

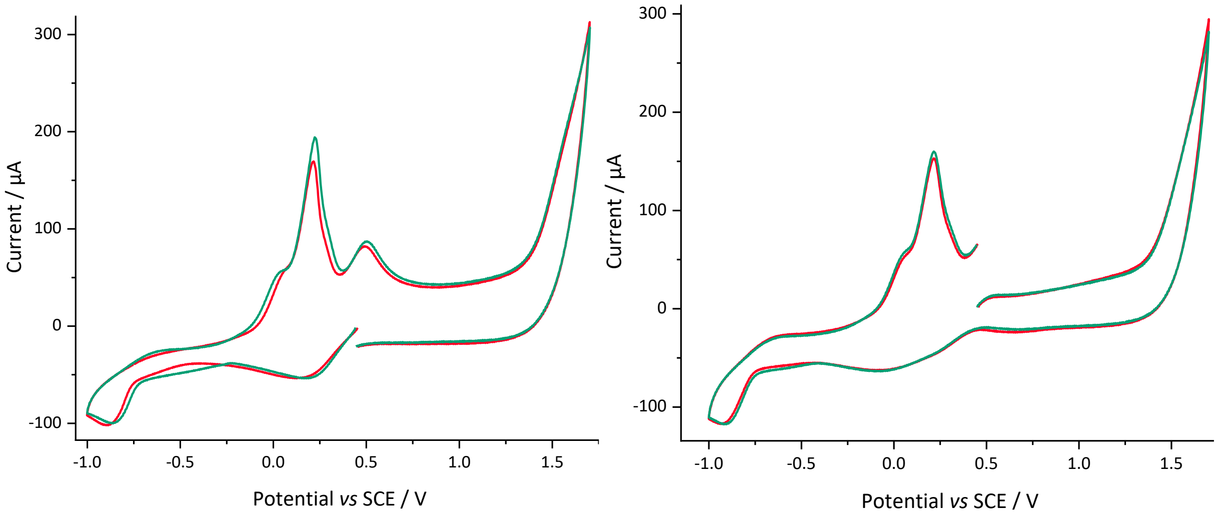

Figure S7. Cyclic voltammogram toward reduction (left) and oxidation (right) potentials of a solution of $CuCl_2$ (2 mM) in MeOH (red line) and in the presence of $PhB(OH)_2$ (10 mM) (green line). Working electrode: glassy carbon (Ø = 3 mm); scan rate: 0.5 V s^{-1}; supporting electrolyte: nBu_4BF_4 (0.3 M) ; recorded at ambient temperature starting at the Open Circuit Potential (OCP).

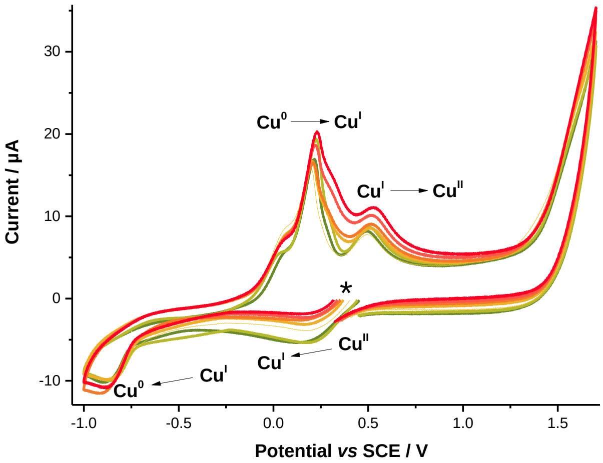

Figure S8. Cyclic voltammetry toward reduction potentials monitoring of the reduction of Cu(II) ($CuCl_2$, 2 mM) to Cu(I) by $PhB(OH)_2$ in the presence of TBAOH (Dark green). After addition of $PhB(OH)_2$ (10 mM, Light green). (Apple green) Addition of TBAOH (5 mM) at t = 0. (Light orange) After 20 minutes. (Orange) After 30 minutes. (Red) After 45 minutes. Working electrode: glassy carbon (Ø = 3 mm); scan rate: 0.5 V s^{-1}; supporting electrolyte: nBu_4BF_4 (0.3 M); recorded at ambient temperature starting at the Open Circuit Potential (OCP).

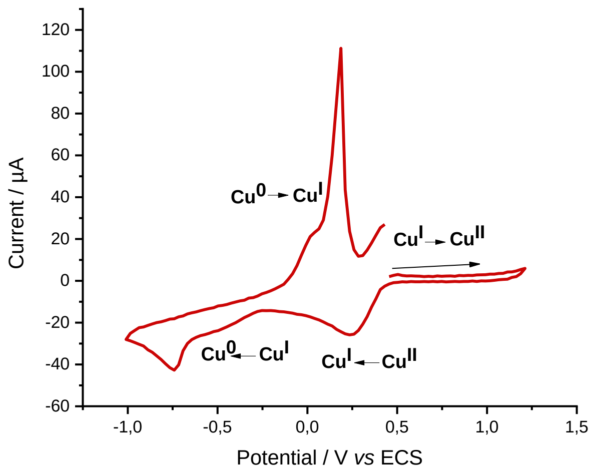

Figure S9. Cyclic voltammogram toward oxidation of a solution of $CuCl_2$ (2 mM) in MeOH. Working electrode: glassy carbon (Ø = 3 mm); scan rate: 0.5 V s^{-1}; supporting electrolyte: nBu_4BF_4 (0.3 M) ; recorded at ambient temperature starting at the Open Circuit Potential (OCP).

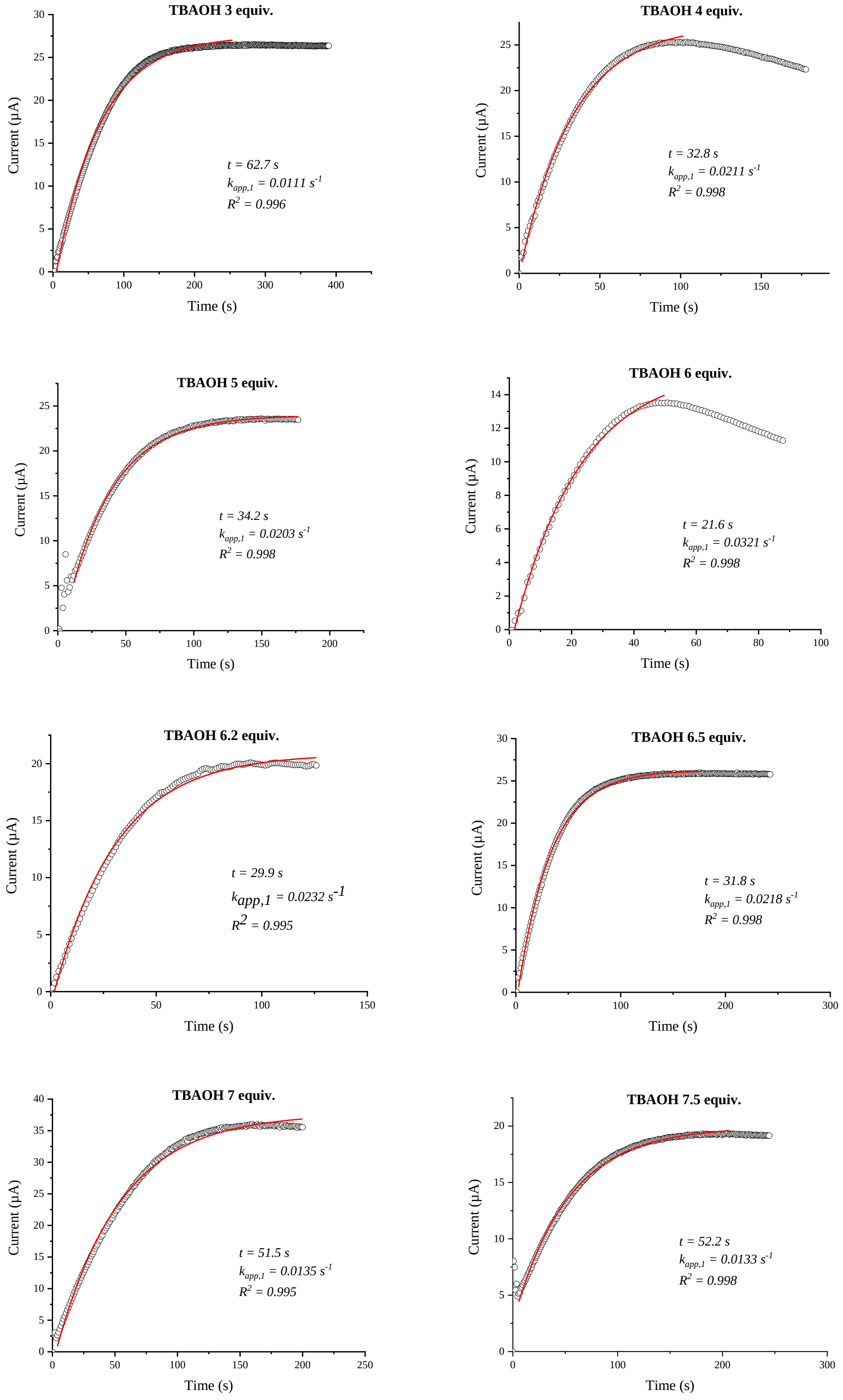

Figure S10. Kinetic monitoring of the reduction of $CuCl_2$ (1 mM) by $PhB(OH)_2$ (10 mM, 10 equiv) in the presence of TBAOH (6 mM, 6 equiv). Working electrode: glassy carbon (Ø = 3 mm); rotation rate: 1000 min^{-1}, imposed potential +0.7 V/SCE; supporting electrolyte: nBu_4BF_4 (0.3 M); thermostat 20 °C, recorded at ambient temperature.

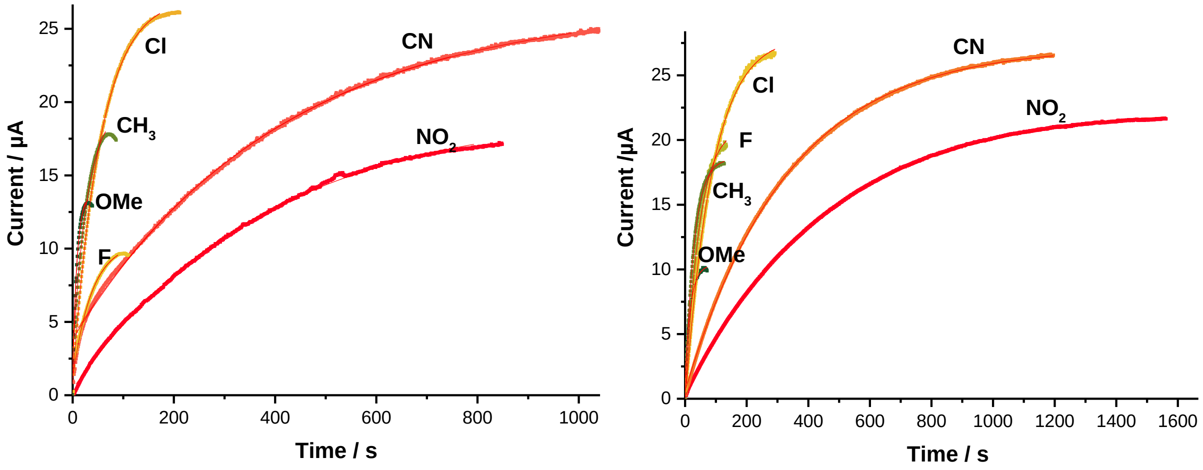

Figure S11. Kinetic monitoring of the reduction of $CuCl_2$ (1 mM) by *p*-X-PhB$(OH)_2$ (10 mM, 10 equiv, X = MeO, CH_3, F, Cl, CN et NO_2) in the presence of TBAOH (4 mM, left / 8 equiv right). Working electrode: glassy carbon (Ø = 3 mm); rotation rate: 1000 min^{-1}, imposed potential +0.7 V/SCE; supporting electrolyte: nBu_4BF_4 (0.3 M); thermostat 20 °C, recorded at ambient temperature.

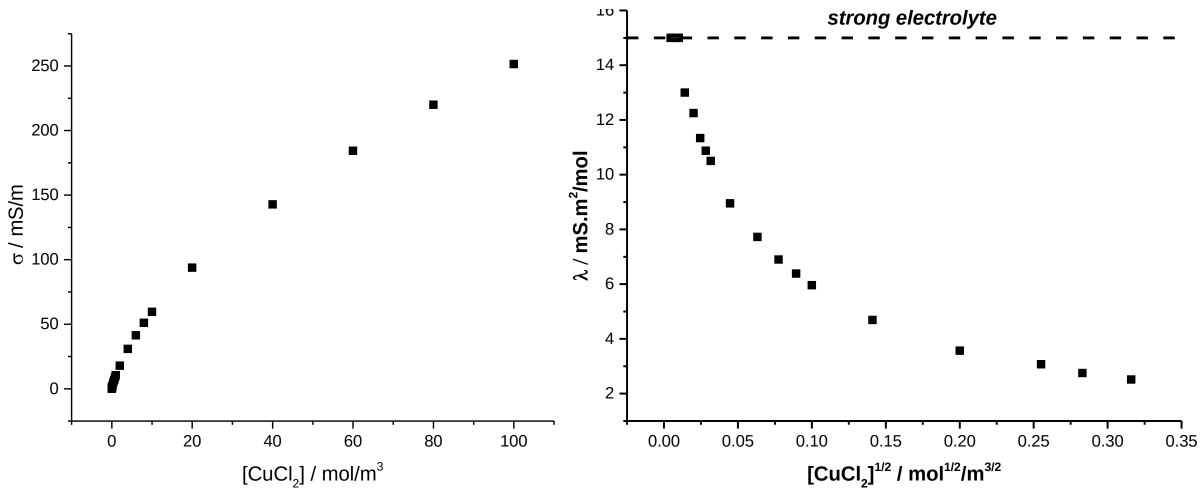

Figure S12. A. Evolution of the conductivity (σ, mS m^{-1}) versus the concentration of added $Cu^{II}Cl_2$ (mol m^3). **B.** Evolution of the ionic molar conductivity of the solution λ (mS m^2 mol^{-1}) versus the square root of the concentration of added $Cu^{II}Cl_2$ (mol$^{1/2}$ m$^{-3/2}$).

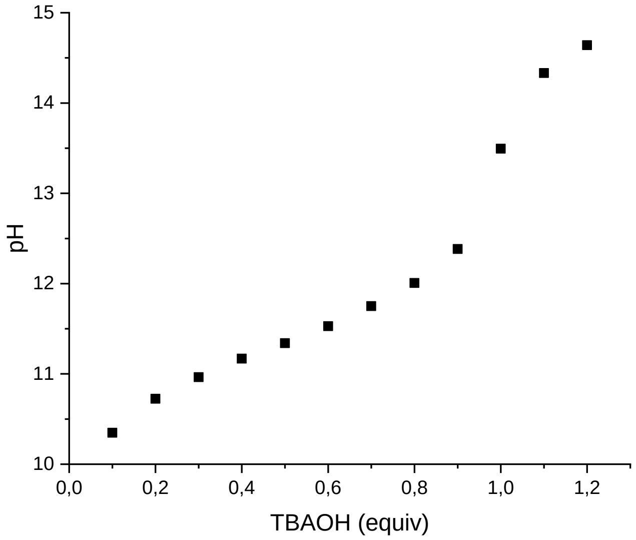

Figure S13. Evolution of the pH during the titration of a solution of phenylboronic acid (10 mmol L^{-1}) by TBAOH (1.0 mol L^{-1} in MeOH). The pKa of the couple $PhB(OH)_2/PhB(OH)_3^-$ can be read 0.5 equiv, pKa = 11.2.

3 B-to-Cu(II) transmetalation

Cartesian coordinates of the structures presented herein can be found in the additional **cartesians.xyz** file.

3.1 Alternative pathways

The mechanism of the first B-to-Cu transmetalation was calculated for X = Cl, MeO and HO. The trends obtained for X = HO are similar to those computed with X= MeO. Δ*G (ΔH)* in kcal mol^{-1}.

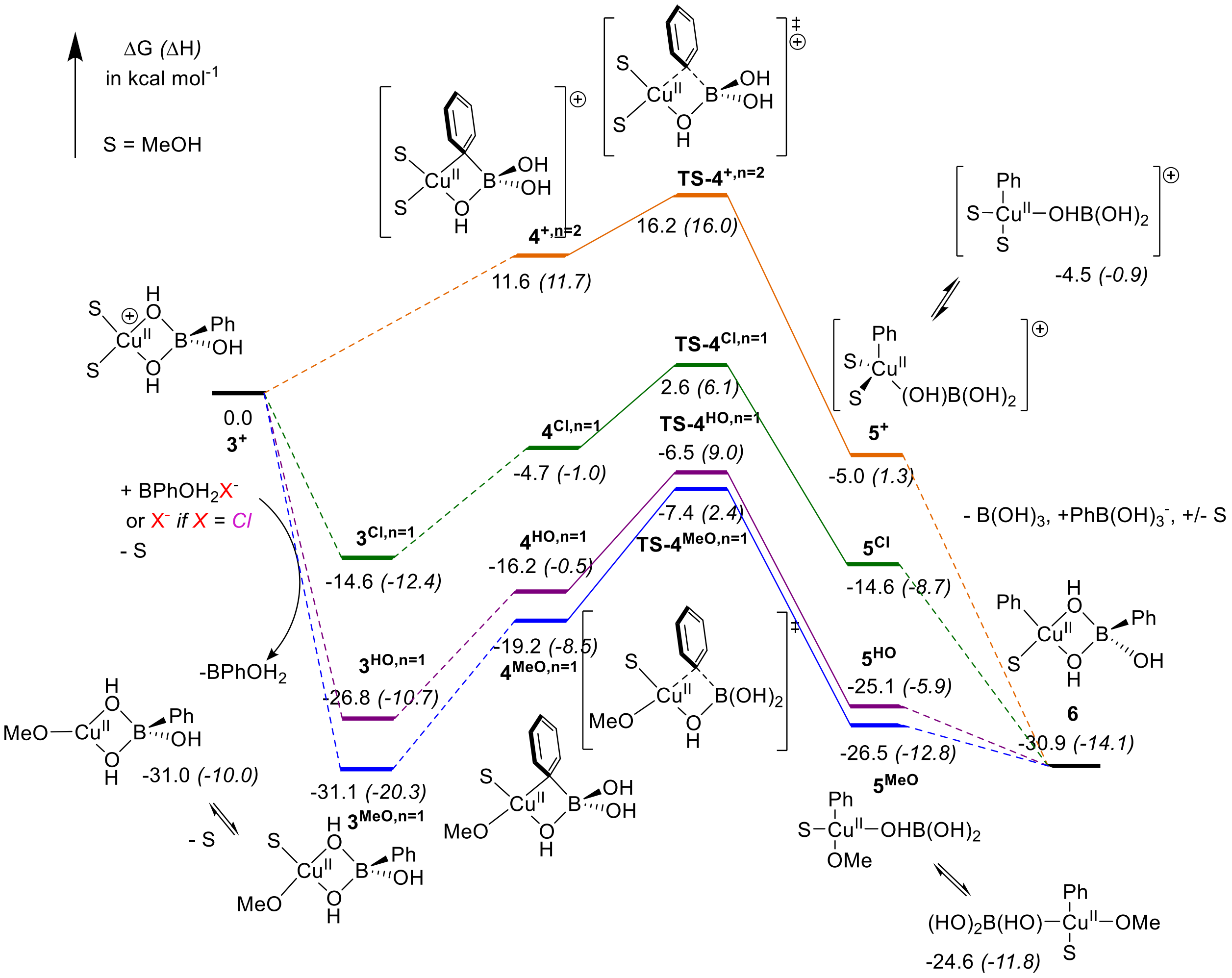

Figure S14. Reaction profiles of the Cu-to-B transmetalation starting for i) the heterobimetallic cationic complex $[(S)_2Cu(\mu\text{-}OH)_2B(Ph)(OH)]^+$ (**3**$^+$) ii) the neutral heterobimetallic intermediate complexes $[S(X)Cu(\mu\text{-}OH)_2B(Ph)(OH)]$ (**3**^{X}) for X = Cl, OH and MeO, computed at the DFT level. Free energies and enthalpies Δ*G (ΔH)* are given in kcal mol^{-1}.

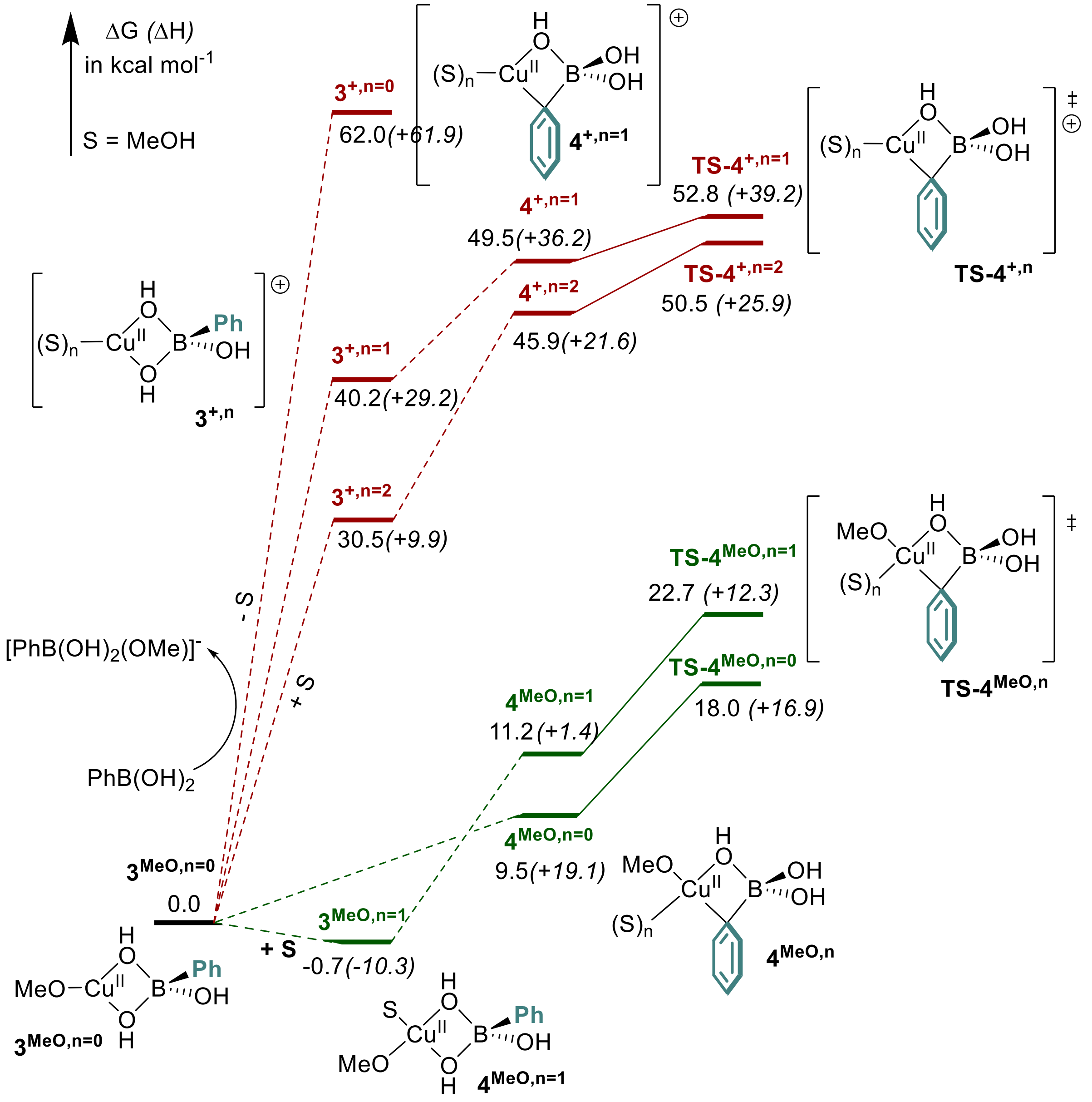

Figure S15. Gibbs free energy calculated at the DFT level for the first boron-to-copper transmetalation involving cationic (red) and neutral (X = MeO, green) intermediates for different solvation states *(i.e. n = 0 to 2 MeOH coordinated to Cu atom)*. Free energies and enthalpies Δ*G* (Δ*H)* are given in kcal mol^{-1}.

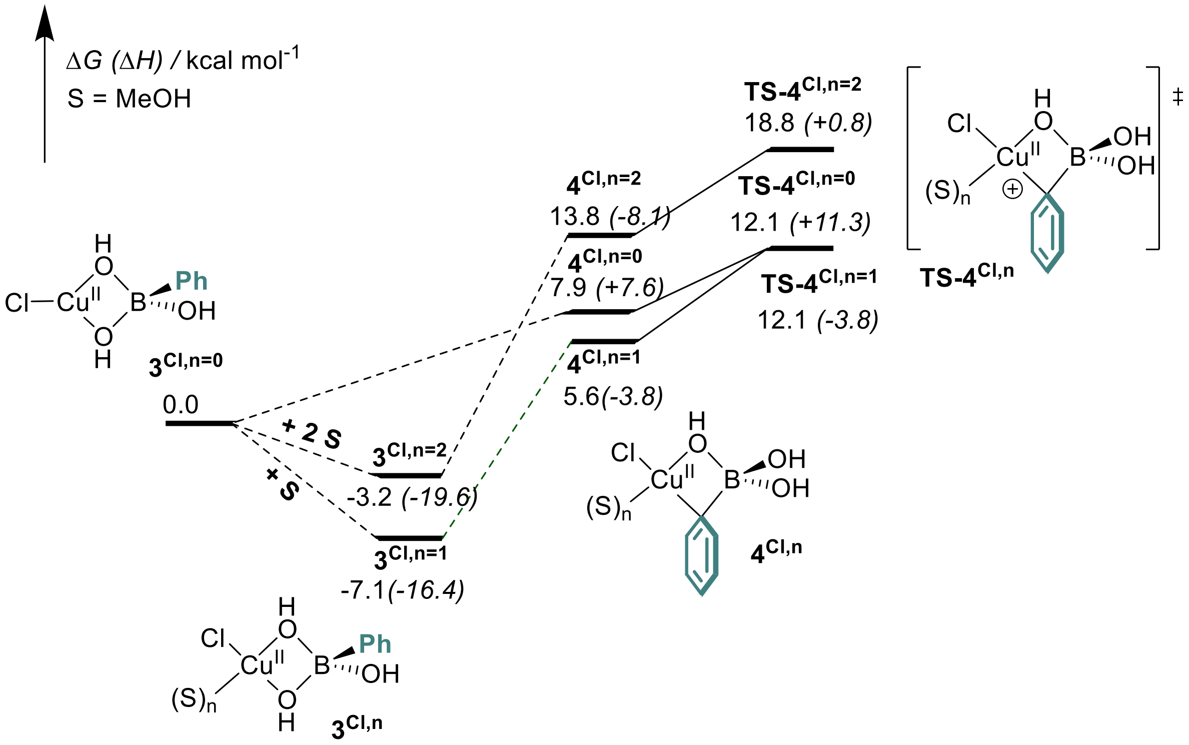

Figure S16. Gibbs free energy calculated at the DFT level for the first boron-to-copper transmetalation involving cationic neutral (X = Cl, n = 0 to 2) intermediates for different solvation states. Free energies and enthalpies Δ*G* (Δ*H*) are given in kcal mol^{-1}.

3.2 NBO analyses of B-to-Cu transmetalation transition states

As can be seen in the following table, all TS share a very similar structure. The higher activation of **TS-4$^{MeO,n=1}$** energy can mainly be attributed to its higher partial charge at the Cu atom. In this regard, the large difference in NBO(Cu) for **TS-4$^{+,n=2}$** and **TS-4$^{Cl,n=1}$** should in principle results in higher activation energy for **TS-4$^{+,n=2}$**, however this is not the case probably because the TS is earlier than **TS-4$^{Cl,n=1}$** (*i.e.* Cu-Ph bond length is relatively few elongated (-0.1 Å) compared to 4^{+}).

Table S1. Comparison of B-to-Cu transition states. Dihedral angles are in degree, distances are in Å, partial charge in atomic unit (1 a.u. = |e^{-}|). The numbers indicated between parenthesis correspond to the bond length variation between **4^{X,n}** and **TS-4^{X,n}** (X = MeOH, Cl or MeO).

	**TS-4$^{+,n=2}$**	**TS-4$^{Cl,n=1}$**	**TS-4$^{MeO,n=1}$**
	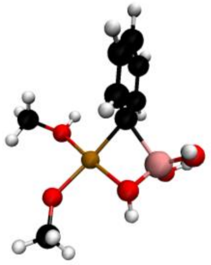	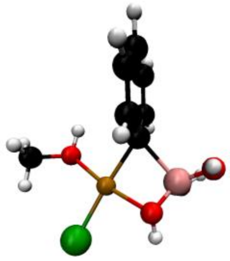	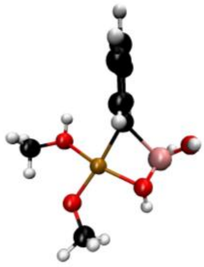
Φ(Cu-C-B-O)	-28.9	-32.2	-31.9
Cu-Ph	2.1 (-0.1)	2.1 (-0.3)	2.1 (-0.4)
Cu-X	2.0 (-)	2.3 (-)	1.9 (-)
Partial charge on Cu	1.315	1.256	1.326

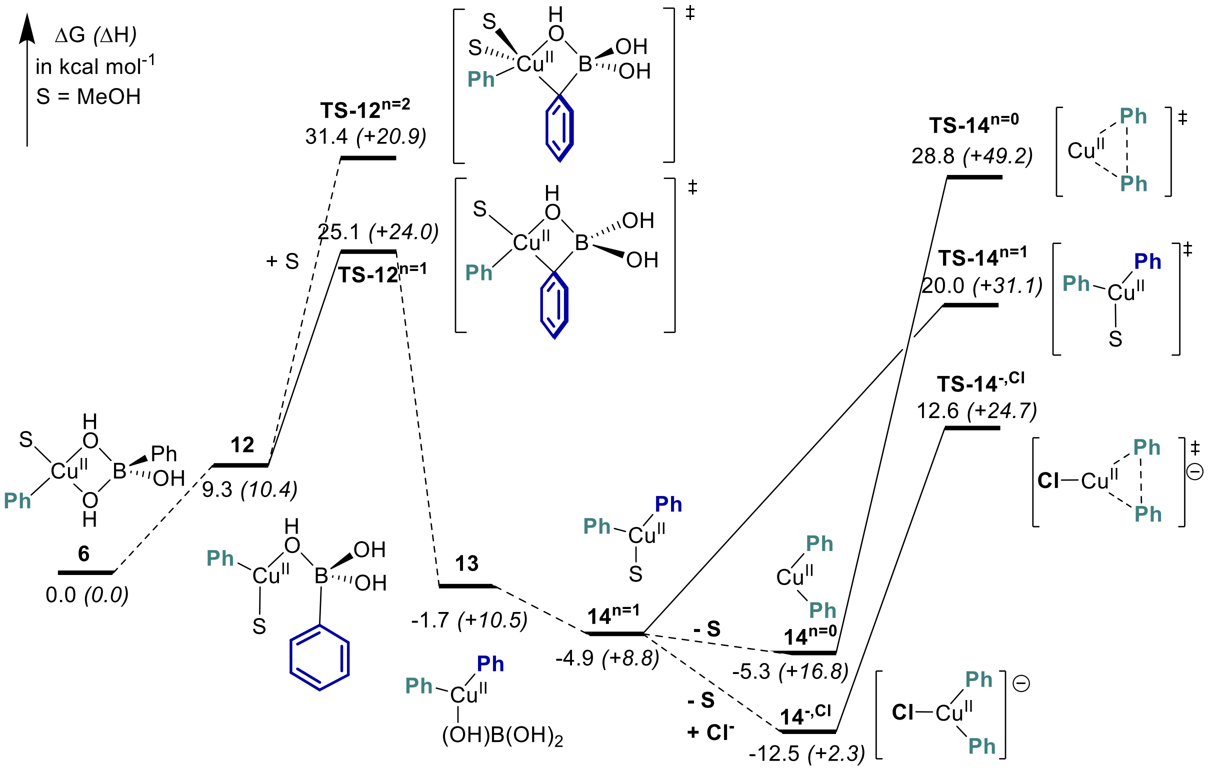

Figure S17. Reaction profiles of the second Cu-to-B transmetalation and following reductive elimination on monomeric organocopper starting for organocopper heterobimetallic complex [(S)(Ph)Cu(μ-OH)$_2$B(Ph)(OH)] (**6**) computed at the DFT level. Free energies and enthalpies ΔG (ΔH) are given in kcal mol^{-1}.

4. Cu-to-Cu transmetalation

Cartesian coordinates of the structures presented herein can be found in the additional **cartesians.xyz** file.

4.1 Speciation of Cu(II)-Cu(II) dimers.

The solvation of Cu(II)-Cu(II) dimers was explored for all combinations of R^1 and R^2. (R^1, R^2 = Cl, OH and/or MeO) These results are displayed in **Table S2**. The coordination of solvent molecules is not favorable; however the first coordination of a MeOH molecule at each Cu(II) center is accessible at room temperature.

Table S2. Formation free energy of Cu^{II}-Cu^{II} dimers with R^1, R^2 = Cl, OH and MeO as bridging groups. Gibbs free energy (Δ***G***, kcal mol^{-1}) and enthalpies (Δ*H*, kcal mol^{-1}) are calculated using the monomers (R^1CuPh) and (R^2CuPh) as a reference. In some cases, one or more MeOH molecules are de-coordinates from Cu during optimization and these structures are quoted *n.d.* The formation of dimers is favored in basic media ($^-$OH or $^-$OMe present).

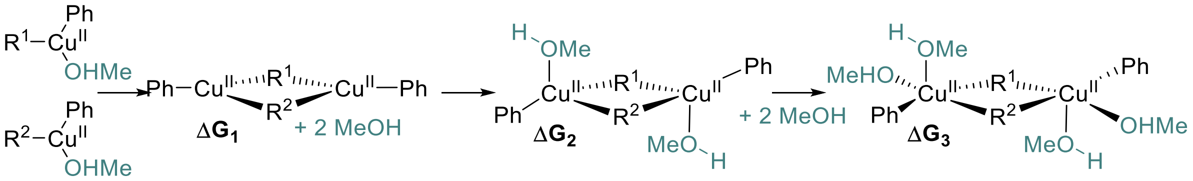

	ΔG_1 (ΔH_1)	ΔG_2 (ΔH_2)	ΔG_3 (ΔH_3)
**R¹, R²** = -Cl	-2.6 (9.6)	1.5 (-5.4)	n.d.
**R¹, R²** = -OH	-21.4 (-14.9)	-15.1 (-24.8)	n.d.
**R¹, R²** = -OMe	-19.9 (-14.5)	-6.3 (-20.6)	n.d.
**R¹** = -Cl | **R²** = -OH	-12.1 (-2.1)	-	11.5 (-18.3)
**R¹** = -Cl | **R²** = -OMe	-12.8 (-3.1)	-	n.d.
**R¹** = -OH | **R²** = -OMe	-21.4 (-14.9)	-	n.d.

The solvation of µ-Ph-µ-R[Cu(II)-Cu(II] species was explored for all combinations of **R^1** and **R^2** and are presented herein. Again, non-solvated dimers are thermodynamically favored, however the first coordination is accessible at room temperature. For **R^1** = **R^2** = Cl, up to 4 MeOH molecules can bind to the dimer (Δ*G* = 6.5, Δ*H* = -27.8 kcal mol^{-1}).

Table S3. Solvation of Cu^{II}-Cu^{II} dimers with R^1 = Ph as bridging groups. Δ***G*** *(ΔH)* in kcal mol^{-1}. In some cases, one or more MeOH molecules are de-coordinate from Cu during optimization and these structures are quoted *n.d.*

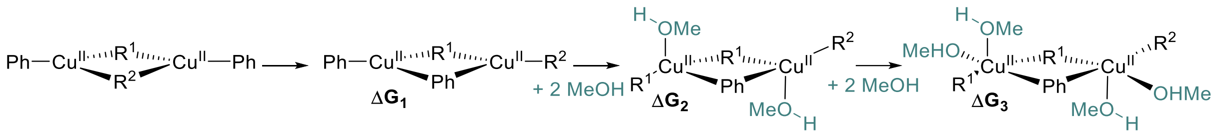

	ΔG_1 (ΔH_1)	ΔG_2 (ΔH_2)	ΔG_3 (ΔH_3)
**R^1, R^2** = -Cl	0.6 (-0.3)	4.4 (-11.6)	n.d.
**R^1, R^2** = -OH	10.3 (9.6)	21.0 (3.1)	n.d.
**R^1, R^2** = -OMe	8.1 (9.0)	n.d.	n.d.
**R^1** = -Cl | **R^2** = -OH	10.1(10.1)	16.1(-0.4)	n.d.
**R^1** = -Cl | **R^2** = -OMe	9.4 (9.3)	16.8 (-0.6)	n.d.
**R^1** = -OH | **R^2** = -Cl	0.3 (0.1)	5.9 (-10.9)	n.d.

4.2. Pathways for Cu-to-Cu transmetalation and reductive elimination.

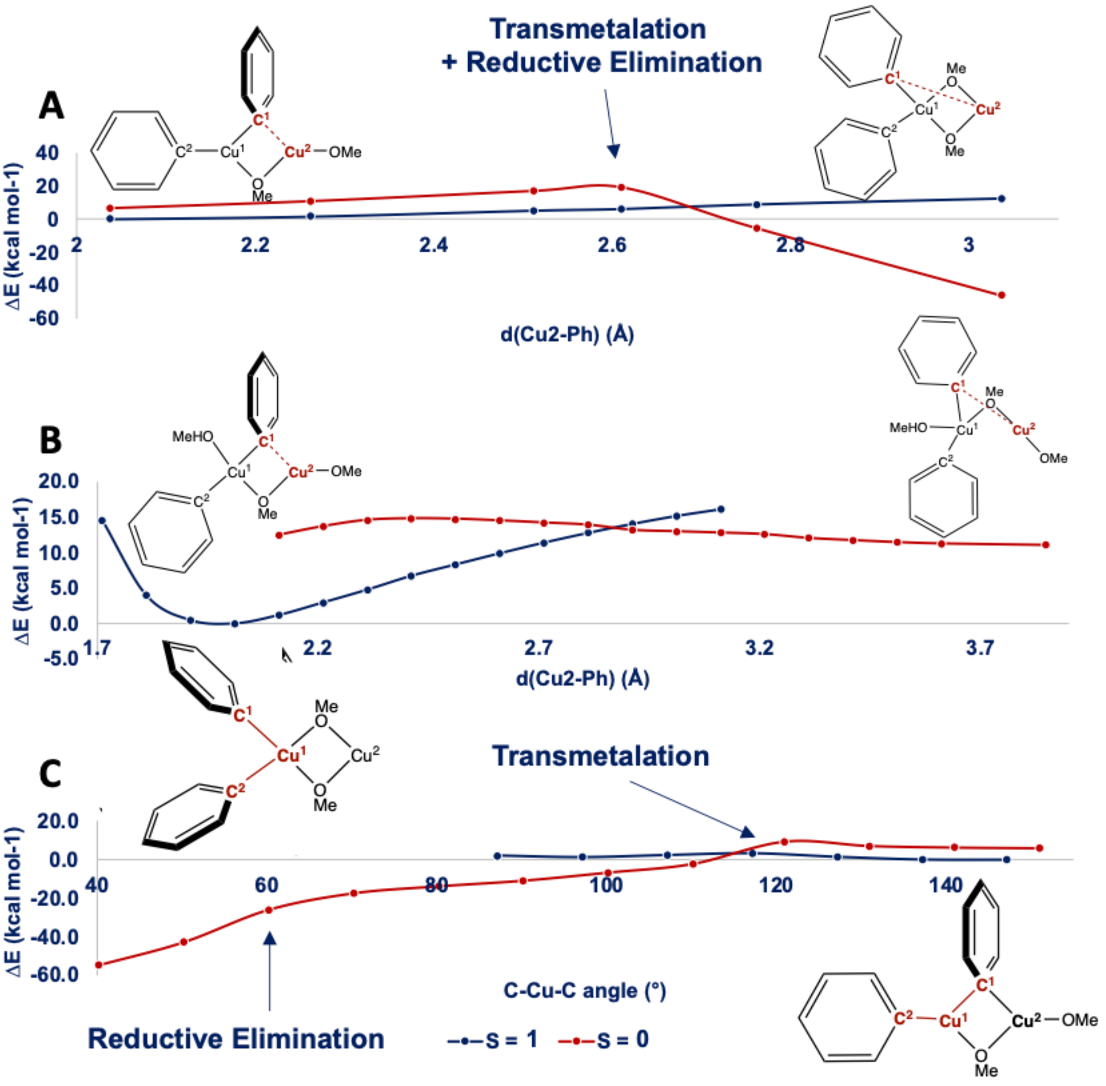

Figure S18. Scans of the triplet and singlet potential energy surfaces over the (μ-(Ph)C_{sp2})-Cu^2 distances for Cu^{II}-Cu^{II} transmetalation *A:* without any MeOH coordinated, *B:* with a "trapping" MeOH and *C:* over the Cu^1-(μ-(Ph)C_{sp2})-Cu^2 angle starting from complexes $\mathbf{11}^{\mathbf{Cl(t)}}$.

The crossing point between the two spin hypersurfaces (S = 0 and S = 1) have been estimated by performing geometry relaxed scans. Initial attempts using the (μ-(Ph)C_{sp2})-Cu^2 distance spontaneously led to the reductive elimination product and the minimum energy crossing point could not be efficiently determined (**Figure S18-A**). When a MeOH molecule is coordinated to Cu^1, the reductive elimination is blocked, and the transmetalation product could be "trapped" by scanning over the μ-(Ph)C_{sp2}-Cu^2 distance (**Figure S18-B**). When the scans are effectuated over the C^1-Cu^1-C^2 angle, the reductive elimination and transmetalation steps can be properly identified (**Figure S18-C**).

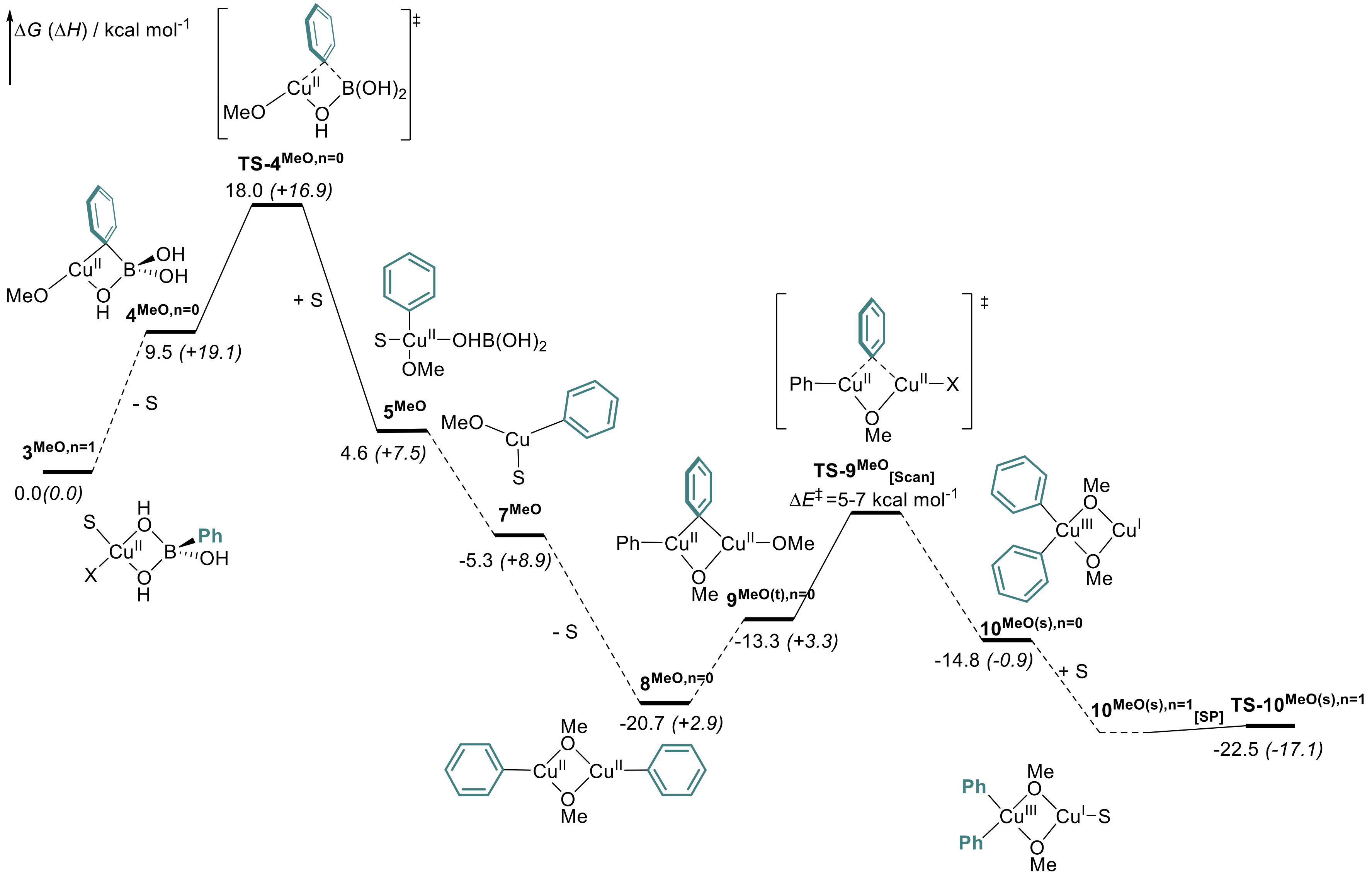

Figure S19. Complete mechanism for homocoupling process computed at the DFT level. Free energies and enthalpies ΔG (ΔH) are given in kcal mol^{-1}.
